# Strategies for enhancing automatic fixation detection in head-mounted eye tracking

**DOI:** 10.3758/s13428-024-02360-0

**Published:** 2024-04-09

**Authors:** Michael Drews, Kai Dierkes

**Affiliations:** Pupil Labs, Sanderstraße 28, 12047 Berlin, Germany

**Keywords:** Fixations, Automatic detection, Eye movements, Head movements, Head-mounted eye tracking, Fixation dataset

## Abstract

Moving through a dynamic world, humans need to intermittently stabilize gaze targets on their retina to process visual information. Overt attention being thus split into discrete intervals, the automatic detection of such fixation events is paramount to downstream analysis in many eye-tracking studies. Standard algorithms tackle this challenge in the limiting case of little to no head motion. In this static scenario, which is approximately realized for most remote eye-tracking systems, it amounts to detecting periods of relative eye stillness. In contrast, head-mounted eye trackers allow for experiments with subjects moving naturally in everyday environments. Detecting fixations in these dynamic scenarios is more challenging, since gaze-stabilizing eye movements need to be reliably distinguished from non-fixational gaze shifts. Here, we propose several strategies for enhancing existing algorithms developed for fixation detection in the static case to allow for robust fixation detection in dynamic real-world scenarios recorded with head-mounted eye trackers. Specifically, we consider (i) an optic-flow-based compensation stage explicitly accounting for stabilizing eye movements during head motion, (ii) an adaptive adjustment of algorithm sensitivity according to head-motion intensity, and (iii) a coherent tuning of all algorithm parameters. Introducing a new hand-labeled dataset, recorded with the Pupil Invisible glasses by Pupil Labs, we investigate their individual contributions. The dataset comprises both static and dynamic scenarios and is made publicly available. We show that a combination of all proposed strategies improves standard thresholding algorithms and outperforms previous approaches to fixation detection in head-mounted eye tracking.

**Supplementary Information:**

The online version contains supplementary material available at 10.3758/s13428-024-02360-0.

## Introduction

The study of gaze behavior has been furnishing insights into human information processing, perception, and other cognitive functions for a long time (Schütz et al., 2011; Rayner, 1978). Today, the use of eye-tracking technology is firmly entrenched in a growing number of scientific fields, such as experimental psychology and neuroscience, but also in applied research, like gaze-based human–computer interaction, market research, and the clinical field (Duchowski, 2002; Klein, 2019).

Video-based eye trackers come in two variants: remote and head-mounted systems (Hansen & Ji, 2010). Remote eye-tracking systems feature one to several cameras for recording the head and eyes of a subject from a distance. Typically, derived gaze estimates are expressed in a world-fixed coordinate system, often relative to a stationary stimulation display. Eye-tracking studies employing remote systems often require subjects to be largely immobile, with head motion being constrained or mechanically prevented altogether.

Head-mounted eye trackers, in contrast, utilize near-eye cameras recording the eyes of a subject from close up. This allows to record gaze data from freely moving subjects under more naturalistic conditions, facilitating the study of social interactions, infancy research, sports research, and more (Pérez-Edgar et al., 2020; Wan et al., 2019). Gaze estimates are commonly expressed in a head-fixed coordinate system relative to the eye tracker itself, often in pixel space of a forward-facing egocentric camera capturing the visual scene. High-acceleration subject movements, headset slippage, and changing lighting conditions represent substantial technological challenges for the robustness of gaze estimation in real-world scenarios. Recent head-mounted eye-tracking systems have increasingly overcome these hurdles, while at the same time becoming more affordable and convenient to use (Baumann & Dierkes, 2023; Kassner et al., 2014; Tonsen et al., 2020).

Humans visually sample their environment by intermittently fixating discrete points (of size smaller than $1-2^{\circ }$, definition depending on research context (Klein, 2019)) in the visual scene, thereby effectively stabilizing visual content on the retina (Schütz et al., 2011). Fixations are separated by saccades, short ballistic eye movements shifting gaze to the next visual target. In order to fixate visual targets in a dynamic world, a number of oculomotor programs are called upon (Schütz et al., 2011): the vestibulo-ocular reflex (VOR) integrates input from the vestibular system to generate stabilizing eye movements counteracting concurrent head movements; the optokinetic nystagmus (OKN) is a periodic eye-movement pattern which stabilizes gaze during motion of the whole visual scene, e.g., during body turns; finally, smooth pursuit (SP) eye movements ensure that our eyes can follow moving targets.

In quantitative eye-tracking studies, fixation statistics often serve as a read-out of overt visual attention. Appropriate metrics, such as the time to first fixation of some target, or the total time spent fixating it, are used to examine human information processing in different contexts, such as reading research, marketing, human–computer interfaces, and in general, to attain insights about the saliency of visual stimuli (Wan et al., 2019; Rayner, 1998). Therefore, the detection of fixations is a crucial step in analyzing gaze data. With humans fixating 3–4 gaze targets per second (Rayner, 2012), in particular automated approaches for fixation detection are desirable.

Standard algorithms for fixation detection are based on thresholding either gaze velocity or gaze dispersion in order to detect periods of stationary gaze (Salvucci & Goldberg, 2000; Andersson et al., 2017). Such methods work well when head motion is limited, as is often the case when using remote eye-tracking systems.

In head-mounted eye-tracking studies, however, fixation detection is significantly more challenging, since gaze stabilization by means of VOR, OKN, and SP often requires fast and large-amplitude movements of the eye. For example, during VOR, eyes can rotate at peak angular speeds as fast as $800^{\circ }\!/\text {s}$, which is comparable to angular speeds during saccades (Sparks, 2002). Under these conditions, standard threshold-based algorithms for fixation detection are prone to misclassification errors when applied naively.

Note that, due to these differences, the very definition of the term *fixation* often depends on the respective research context (Hessels et al., 2018). Research studies performed in static settings with head-stabilized participants tend to define fixations in a purely oculomotor way, i.e., via the absence of eye-in-head motion. Since gaze stabilization in freely moving subjects generally involves a complex combination of the aforementioned active mechanisms, studies using head-mounted eye trackers, instead, often employ a functional definition of what is considered a fixation (Hessels et al., 2018). Here, we adopt this approach and explicitly consider any eye movement that is geared towards the stabilization of a visual target on the retina as part of a fixation.

### Contributions

The main focus of our work is to devise a method for automated fixation detection, which – after suitable tuning of algorithm parameters for a given head-mounted eye tracker – works reliably for a broad set of static and dynamic real-world use cases. To this end, we extend standard threshold-based algorithms and suggest three strategies for enhancing the detection of functional fixations in dynamic scenarios.

First, to reliably detect fixations also during head motion, we introduce a pre-processing stage which compensates for gaze shifts that are coherent with the motion of visual content in the scene camera as estimated by optic flow. We show that this head-motion compensation stage improves fixation detection in head-mounted eye-tracking data using both velocity-based as well as dispersion-based algorithms.

Second, we show that adaptively adjusting the velocity- or dispersion-threshold, respectively, according to the magnitude of optic flow in the camera image, serving as a proxy for head-motion intensity, further improves fixation detection.

Third, we demonstrate the benefit of coherently tuning all parameters determining detection performance by means of a global optimization. In particular, this encompasses parameters of event-based post-processing filters.

We quantify the individual contribution of each strategy and show that their combination enables fixation detection even in highly dynamic real-world scenarios, surpassing other algorithms in terms of relevant performance metrics. Our evaluation is based on a new dataset for fixation detection in head-mounted eye tracking, comprising recordings and corresponding manual ground-truth annotations of fixations in a wide range of both dynamic as well as static settings. We publish this dataset alongside this study.

In summary, we propose an approach for fixation detection that is suitable to be used in conjunction with head-mounted eye-tracking systems in a variety of different research contexts.

## Related work

Our results are related to prior work on automated fixation detection, both in remote as well as in head-mounted eye-tracking systems.

### Fixation detection in static settings

Standard algorithms for automated fixation detection can be categorized into velocity- and dispersion-based approaches, respectively (Salvucci & Goldberg, 2000).

The basic velocity-based method classifies individual samples of the gaze signal as belonging either to a fixation or not, depending on whether gaze speed is below or above a given threshold. The principal idea of this I-VT algorithm (**I**dentification by **V**elocity **T**hreshold) has been further elaborated in numerous studies. For example, variants have included noise-adaptive thresholds (Engbert & Mergenthaler, 2006; van der Lans et al., 2011), or multiple thresholds resolving additional types of eye movements, such as smooth pursuit (Larsson et al., 2015; Komogortsev & Karpov, 2012) and/or post-saccadic oscillations (Nyström & Holmqvist, 2010; Dar et al., 2020).

Similarly, dispersion-based methods (I-DT algorithms) are based on thresholding an estimated value of the spread of consecutive gaze points within a sliding window (Salvucci & Goldberg, 2000; Veneri et al., 2011).

Next to what we refer to as standard algorithms, a range of other methods have been proposed over the years. Among those are approaches relying on Kalman filters for inference of the underlying state of the oculomotor system (Komogortsev & Khan, 2009), or hidden Markov models (Komogortsev et al., 2010) to model the gaze data as a probabilistic sequence of eye-state transitions.

More recently, machine learning has been applied to event detection in eye-tracking data. This includes Bayesian approaches for probabilistic inference of the eye state (Kasneci et al., 2015; Santini et al., 2016), clustering algorithms for noise-robust detection of gaze shifts (Hessels et al., 2017), or the application of classic machine learning models, e.g., random forests, for predicting fixations based on a multi-dimensional set of gaze-related features (Zemblys, 2017; Zemblys et al., 2018). Typically, eye-tracking data are processed sample-wise, i.e., gaze samples are treated as statistically independent from each other. Another interesting approach is, therefore, the use of neural networks to detect eye movements in time series of eye-tracking data, which has been explored using both convolutional neural networks (CNNs) (Hoppe & Bulling, 2016) as well as recurrent network architectures(Zemblys et al., 2019; Startsev et al., 2019).

Note that all of the aforementioned methods have been developed for static viewing conditions, often using remote eye trackers and assuming little to no head motion. The underlying assumption of fixations corresponding to time periods of approximately stationary gaze, however, does not generally hold when dealing with data from head-mounted eye trackers and in highly dynamic settings.

### Fixation detection in head-mounted settings

Only a few studies have looked at automated fixation detection in head-mounted eye tracking. Generally, a functional definition of the term *fixation* as a period of retinal image stabilization is adopted in this context (Hessels et al., 2018; Patla & Vickers, 1997; Franchak et al., 2011; Steil et al., 2018). As noted earlier, head-mounted eye trackers report gaze in a head-fixed coordinate system, reflective of eye-in-head motion. Dissecting stabilizing eye movements further into VOR, OKN or SP is challenging in this setting, as such discrimination necessitates correlating eye-in-head motion with gaze in a world-fixed coordinate system and/or an independent quantification of head motion.

One study achieves this in a virtual reality (VR) setup using head-mounted displays (HMDs) (Agtzidis et al., 2019). Working in VR allows for gaze position to be expressed both in a subject-centered as well as a world-centered coordinate system, rendering a classification of the different types of stabilizing eye movements feasible. Here, we do not assume estimates of gaze other than in a head-fixed coordinatesystem.

To go beyond the isolated analysis of gaze dynamics, other approaches have included visual information from the egocentric scene camera instead. Kinsman et al. (2012) estimate ego-motion from global image shifts between scene camera frames via image cross-correlation. This ego-motion signal is used to transform eye-in-head gaze velocity and express it in a world-centered reference frame. Fixation detection is then implemented using an I-VT algorithm on this pre-processed signal. While this is conceptually very similar to our head-motion compensation stage, the study provides evaluation only on simulated data and a thorough comparison with other methods using transferable performance metrics is missing.

Anantrasirichai et al. (2016) build on the idea that while performing a specific task, i.e., locomotion in outdoor terrains, subjects tend to fixate on task-relevant aspects of their surroundings, e.g., stones lying on the path in this case. More specifically, they combine hand-crafted features reflecting gaze dynamics with CNN-features extracted from image patches centered around the gaze point and train an SVM-classifier to distinguish fixation from non-fixation samples. Their work is explicitly aimed at improving automated fixation detection in head-mounted eye trackers with a low sampling rate (30 Hz) and is dependent on task-specific aspects of fixation statistics. Here, we aim at an approach of more general applicability and do not make the assumption of low sampling rate.

Finally, embracing a functional definition of the term fixation, Steil et al. (2018) propose to detect fixations based on the visual similarity of gaze targets between frames. Using a patch-similarity network, i.e., a CNN pre-trained to estimate the visual similarity of local image patches, they construct a similarity score for pairs of gaze-centered image crops from consecutive camera frames. This similarity score is then used as input to a standard threshold-based algorithm, thus effectively detecting periods of visual similarity with respect to the gaze target. Evaluating this approach on head-mounted eye-tracking data, the authors show increased performance and robustness of this method in comparison with velocity- and dispersion-based standard algorithms. However, it is not clear how well such deep-learned image similarity metric generalizes to other datasets, using different scene cameras, or containing video content that differs from the data used to train the patch-similarity network.

Generally, a quantitative comparison between existing approaches is hampered by the use of different performance metrics in each study. Over the last years, however, the field of fixation detection seems to be converging towards the use of event-based evaluation metrics, e.g., an event-based F1 score, which can be calculated after matching predicted events to ground-truth events (Hooge et al., 2018; Startsev et al., 2019; Zemblys et al., 2019; Startsev & Zemblys, 2022). In our study, we adopt this approach to systematically benchmark different methods for fixation detection in head-mounted eye tracking.

### Event-based post-processing

Since sample-wise classification is prone to yield fragmented outputs in the presence of noise, event-based post-processing filters are commonly used to remove physiologically implausible event detections. Effectively, for sample-based algorithms, event-based post-processing is the only way to exploit temporal correlations in the data. One common step is to remove fixation fragments that are too short to be considered physiological fixations (Hooge et al., 2022; Salvucci & Goldberg, 2000; Andersson et al., 2017). Others additionally merge neighboring fixations whenever the gap separating them falls below fixed threshold values for minimum saccade duration and minimum saccade amplitude (Olsen, 2012).

Interestingly, while most methods in eye-movement detection are composed of a sample-wise classifier stage followed by some kind of event-based post-processing stage, until recently almost no attention has been given in the literature to the role of this post-processing stage. The parameters of post-processing filters for suppressing unphysiological predictions are usually chosen ad hoc and depending on research context. For example, minimum saccade amplitude was defined as $1.0^{\circ }$ when studying fine-grained eye movements during a visual search task in head-fixed participants (Kemner et al., 2008), while a value of $3.0^{\circ }$ was used when studying interactive behaviors like a racquet game in virtual reality (Diaz et al., 2013). Minimum fixation length can range from 40 (Hessels et al., 2016) to 240 ms(de Barbaro et al., 2011).

Importantly, a recent study found the differences between alternative sample classifiers before post-processing to become significantly smaller when compared after event-based post-processing (Hooge et al., 2022). This implies that the optimal choice of post-processing parameters plays a crucial role in modifying the output of the sample-based classification stage.

In our work, therefore, we pay particular attention to the contribution of event-level post-processing to algorithm performance. More specifically, we investigate how overall performance depends on the coherent tuning of all algorithm parameters.

### Head-mounted eye-tracking datasets

In order to evaluate fixation-detection algorithms, annotated datasets are required. In the context of head-mounted displays and virtual reality, several datasets have been released alongside corresponding stimulus material, gaze-tracking data, and annotated ground-truth events for different types of eye movements (David et al., 2018; Rai et al., 2017; Agtzidis et al., 2019). While subjects were free to move their head in most of these experiments, usually they could not freely move within the virtual reality or interact with it in a natural way. Thus, these experiments differ from real-world tasks as recorded by head-mounted eye trackers.

Only a limited number of prior studies on fixation detection using head-mounted eye trackers have included publicly available datasets. The *MPIIEgoFixation* dataset (Steil et al., 2018) amounts to 25 min of eye-tracking data, including gaze estimates and ground-truth fixation labels. However, due to the lack of scene-camera footage, optic flow cannot be calculated for these recordings, precluding it of being used for the current study. The *Gaze-in-wild* dataset (Kothari et al., 2020) contains more than 2 h of annotated eye-tracking data recorded during real-world tasks. The head-mounted eye tracker used for recording *Gaze-in-wild* employs a geometric 3D eye model for gaze estimation and requires calibration prior to each experiment. While being useful in its own right, this approach to gaze estimation has been shown to be hampered by headset slippage, requiring frequent recalibration, as well as by data loss, e.g., due to challenging lighting conditions.

Here, we decided to employ more recent eye-tracking technology, which is geared towards overcoming many of these limitations. More specifically, we chose to record a new dataset using the Pupil Invisible glasses by Pupil Labs (Tonsen et al., 2020), a calibration-free eye tracker designed to be worn not only during screen-based lab experiments but in particular also during active behaviors and in real-world settings. Our dataset is publicly available and comes with manually annotated ground truth events (for details see next section).

## Dataset

For tuning and evaluation of our approach, we recorded a dedicated dataset (available at https://osf.io/8en9v/) using the head-mounted Pupil Invisible glasses by Pupil Labs (Tonsen et al., 2020). They feature an egocentric scene camera with a resolution of 1080$\times$1088 pixels and a frame rate of 30 Hz. Two near-eye IR cameras record the left and right eye, respectively, at a frame rate of 200 Hz. Gaze estimation is performed by means of a machine-learning pipeline employing convolutional neural networks. More specifically, it processes concurrent left and right eye images and reports final gaze estimates relative to the pixel space of the scene camera.

Pupil Invisible does not require a calibration prior to its use. It delivers gaze estimates with a mean subject error of $\sim 5.5^{\circ }$ as measured over a wide field of view, different slippage configurations, as well as indoor and outdoor recording scenarios (Tonsen et al., 2020). In particular, the gaze-estimation algorithm is robust to perturbations such as headset slippage and varying lighting conditions, including being used in direct sunlight.

Our dataset consists of two parts: a dynamic and a static subset. The dynamic dataset comprises recordings of subjects performing active tasks in complex and dynamic real-world environments. Specifically, we collected a total of 14 min of eye-tracking data from 16 subjects with behavioral tasks such as searching for a specific product in a supermarket, free exploration of urban environments, playing an instrument, or driving a car. The total time recorded varied between subjects. An exact mapping from subject to individual recordings, including relevant metadata, can be found in our online data repository. Note, some recordings contain dynamic external objects, such as cars or other people passing by. While fixations on such objects do fall under our fixation definition (see below), we observed relatively few such events.

For the static dataset, subjects were tasked to passively observe a set of videos on a computer screen, thus emulating screen-based laboratory experiments. No head-restraints were used, but subjects were instructed to move their head as little as possible. The video stimuli displayed, however, contained dynamically changing image content. More specifically, a screen recording of a computer game, an abstract moving-dot stimulus, and a movie scene were shown to all subjects. The static subset comprises a total of 7.5 min of eye-tracking data from five subjects, with 1.5 min per subject.

For both subsets, data were collected by selecting relevant periods, representative of the task and of duration less or equal than 1 min. Gaze estimates were used as reported by the eye tracker, i.e., no further pre-processing was performed. In total, the dynamic set consists of 24 and the static subset of 15 individual recordings. For cross-validation, we generated five folds by randomly selecting individual recordings for a given validation fold, while approximately balancing the ratio of static and dynamic recordings for each fold.

The dataset, as well as information about the exact data splits, is published alongside this study, and includes scene-camera videos, gaze estimates, and other derived quantities necessary for reproducing our results.

### Ground-truth annotations

#### Fixation definition

The annotation of ground-truth fixations was guided by a *functional* definition of the term fixation. Specifically, we consider any period during which a gaze target is stabilized on the retina as a fixation. Micro-saccades, i.e., jerk-like eye movements occurring during fixations (Otero-Millan et al., 2008; Martinez-Conde et al., 2004), are explicitly subsumed under this definition, meaning that fixations are not broken up by micro-saccades. In practice, we regard abrupt changes in gaze position smaller than $2^{\circ }$ in amplitude and shorter than 50 ms as micro-saccades.

**Fig. 1:**
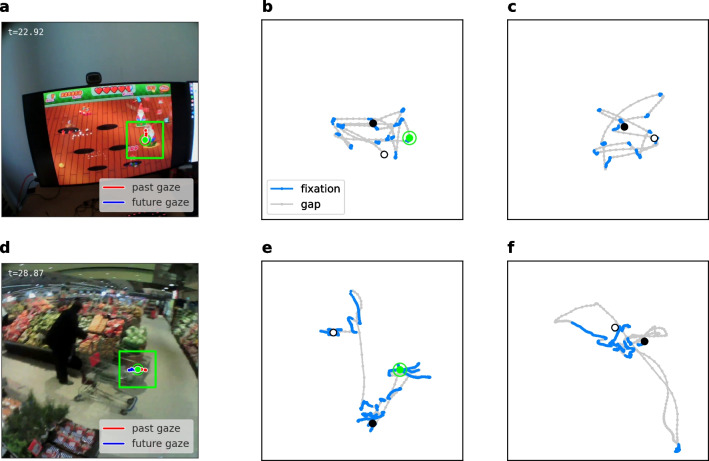
Example gaze trajectories. **a** Snapshot of one frame during a fixation in a recording from the static dataset (see also Suppl. Video 1). The *green dot* marks current gaze position. Past and future gaze position around the time of the snapshot (± 0.33 s) are highlighted in *red* and *blue*, respectively. However, they are partially masked by the *green dot*, since gaze position is stationary during fixations in the static dataset (without head movements). **b** Five-seconds-long gaze trajectory from the same recording as in (a), where the $\bullet$ marks the first frame and $\circ$ the last frame of the sequence. Gaze position from the snapshot in (a) is highlighted in *green*. **c** A different 5-s-long gaze trajectory from the same recording as in (a). (**d**–**f**) Same as (a-c), but in a recording from the dynamic dataset (with head movements; see also Suppl. Video 2). In this frame (at a time of 28.87 s), the subject is fixating on the corner of the shopping cart while turning their head towards the right

**Fig. 2:**
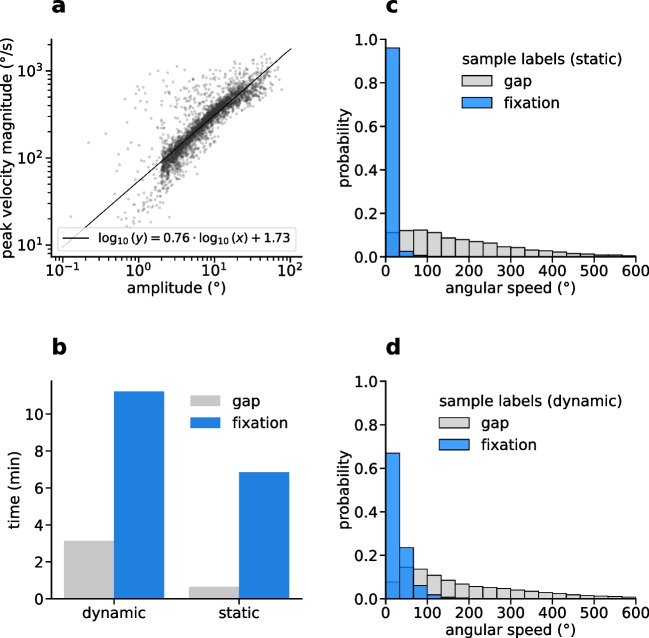
Dataset descriptive statistics. **a** Scatterplot of amplitude vs. peak velocity magnitude of all events labeled as “gap” in the dataset, in log-log coordinates. The relationship resembles the main sequence of saccades, which can be described as a power-law (*black line* shows a corresponding linear fit to the data in log-log space). **b** Accumulated duration of annotated “gaps” and “fixations” in the dataset. Subjects spent overall around 83% of the time fixating, 78% for the dynamic dataset, 91% for the static dataset. **c** Histograms over angular speeds for individual samples in the static dataset, according to the event label at the time of each sample. Histograms for fixations and gaps are normalized separately, so that the area under each histogram is 1. Speed distributions of fixations and gaps overlap for small velocities. **d** Same as in (c), but for the dynamic dataset. For the dynamic dataset, overlap between the speed distributions of fixations and gaps is higher than in the static dataset

#### Annotation procedure

Fixations were annotated manually, according to the definition given above. For this purpose, we developed custom software (Suppl. Fig. 1). It displayed gaze position and gaze velocity, as well as the current scene-camera frame with an overlay of the past, current, and future gaze estimates (within $\pm 33$ ms of the current time). Fixations were annotated by marking their start and end time points. In practice, whenever the gaze trajectory in scene-camera image space was locked for a period of time to the movement of the local image content, the full period was annotated as a fixation.

For example, when gaze was moving in unison with the whole image, as is characteristic for VOR eye movements during head-turns, this was marked as a fixation. Similarly, a fixation was, e.g., also annotated, whenever gaze was directed towards an independently moving target in the image and tracking apparent object motion over time. However, such smooth pursuit events, although not actively avoided during data collection, were found to be relatively rare.

Gaze-estimation errors can lead to offsets between gaze estimates and gaze-followed objects in the image. The annotator still marked a fixation, as long as it was contextually clear that the gaze point was co-moving with local image structures in the vicinity.

One intricacy in annotating the data was the fact that the scene camera view is updated at 30 Hz, while the gaze signal is sampled at 200 Hz. We observed that it was challenging for a human annotator to reliably judge whether gaze remained fixated on some visual target when its position was updated 6–7 times more frequently than the camera image. To alleviate this issue, we used Real-Time Intermediate Flow Estimation (RIFE) to temporally upsample the scene camera video to a frame rate of 240 Hz (Huang et al., 2020). RIFE utilizes deep neural networks to synthesize intermediate frames in between consecutive video frames (see Suppl. Video 3). Note that these synthesized videos were used exclusively during annotation to establish high quality ground-truth labels. Annotations were generated by one of the authors of this study. Finally, in order to remove micro-saccades (Otero-Millan et al., 2008) in accordance with our fixation definition, we merged fixations which were separated by gaps smaller than $2^{\circ }$ in amplitude and shorter than 50 ms as a post-processing step.

In Fig. 1, example gaze trajectories are shown together with ground-truth annotations. For the static dataset (Fig. 1a-c), we found typical trajectories characterized by tight clusters during fixations, which were separated by ballistic saccades. Such trajectories have often been described in head-fixed experimental setups (Otero-Millan et al., 2008).

In contrast, for the dynamic dataset (Fig. 1d-f), we found that gaze points belonging to the same fixation did not always cluster. For example, whenever the subject was stabilizing gaze on a target while simultaneously moving their head (e.g., at a time of 28.8 s in the example; see also Suppl. Video 2), effectively performing VOR, gaze points were approximately arranged along lines in scene camera space.

### Dataset descriptive statistics

While we did not introduce a dedicated label for saccades in our dataset, gaps between fixations to some extent can be interpreted as such. This is supported by the finding that peak magnitude of gaze velocity during gaps scales according to a power law with gap amplitude, the latter being defined as the angular difference between start and end point (Fig. 2a). This closely resembles the main sequence relationship which has been described for saccades previously (Otero-Millan et al., 2008). However, in some cases, gaps can also merely represent periods of increased difficulty in annotating the data, e.g., due to the presence of noise and very rapid head motion.

When analyzing the total time spent fixating versus the accumulated time of gaps, we found a difference between the static and dynamic dataset (Fig. 2b). The proportion of gaps is increased for the dynamic dataset (22%) in comparison with the static dataset (9%). This difference might be partially due to the greater difficulty of annotating fixations in the dynamic dataset. Interestingly, however, this ratio is consistent with results reported previously in a similar setting (Steil et al., 2018).

On the sample-level, fixations and gaps are not clearly separated by angular gaze speed (Fig. 2c,d). While the speed distribution of gap samples is clearly skewed towards higher speeds up to $600^{\circ }\!/\text {s}$ and more, there is a substantial overlap with the speed distribution of fixational samples for speeds below $100^{\circ }\!/\text {s}$. This is especially the case for the dynamic dataset, emphasizing the need for more elaborate methods of fixation detection than mere velocity-based thresholding.

## Methods

In this section, we will expound the proposed three strategies for improving standard I-VT and I-DT algorithms when used in conjunction with head-mounted eye trackers. To this end, we first introduce the metrics used for gauging algorithm performance in later sections. We then describe the pertinent versions of the standard I-VT and I-DT algorithm, including an event-based post-processing stage. Next, we deal with each strategy (head-motion compensation, adaptive thresholding, coherent tuning of all algorithm parameters) in turn. We finish this section by briefly commenting on other fixation-detection algorithms, which will be part of our evaluation.

### Performance evaluation

Different metrics have been proposed in the literature for the evaluation of fixation-detection algorithms. Earlier work often reports average statistics, such as the mean number of fixations detected, as well as their mean duration and standard deviation (Komogortsev et al., 2010; Nyström & Holmqvist, 2010; Kinsman et al., 2012; Andersson et al., 2017; Hessels et al., 2017). While such statistics are useful for a high-level plausibility check of results, they do not provide a metric that is sensitive to the precise temporal structure of sequences of fixations and saccades.

#### Cohen’s kappa

To evaluate the quality of predicted fixations against ground-truth labels on a sample-level, a common measure is the Cohen’s kappa metric (Larsson et al., 2015; Santini et al., 2016; Andersson et al., 2017; Zemblys et al., 2018). Cohen’s kappa takes into account disproportionate class frequencies, which is appropriate for eye-tracking data (see Fig. 2b).

More specifically, we calculate Cohen’s kappa as

$$\kappa _{samples}=\frac{p_0 - p_c}{1 - p_c},$$

where $p_0$ is the sample-level accuracy of the algorithm, and $p_c$ is the average accuracy of a random baseline with representative class probabilities. Cohen’s kappa takes on values between 1 and -1, where 1 means perfect agreement between prediction and ground truth, and 0 means no more agreement than what would be expected by chance.

**Fig. 3:**
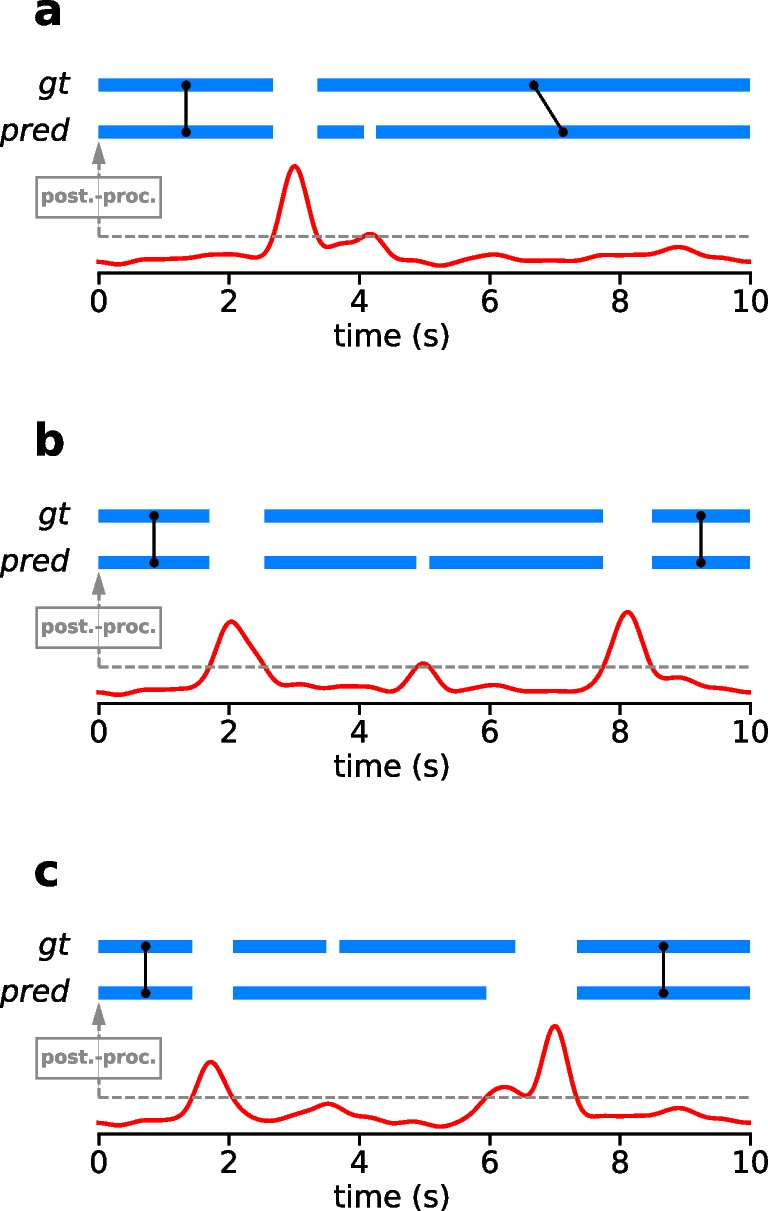
Event-matching for the event-level F1 score. Two examples to illustrate event matching between ground-truth annotations and predicted sequences. *Top row*: ground-truth (gt) and predicted (pred) event sequences. Fixations are highlighted in *blue*. *Bottom row*: angular gaze speed (*red*) and magnitude of velocity threshold (*dashed grey line*) underlying the predictions for each example. **a** Example 1: Ground-truth fixations are matched with the predicted fixations that have the largest overlap in terms of intersection-over-union score (IoU). One predicted fixation cannot be matched, and, therefore, counts as a false-positive. **b** Example 2: The predicted event sequence is fragmented due to a small peak in the gaze speed, which has not been annotated as a saccade or gap in the ground-truth. Hence, predicted fixations do not fulfil the minimum criterion of 70% IoU score. The unmatched fixation events count as two false-positive predictions, and the unmatched ground truth fixation as one false-negative. **c** Example 3: Gaze speed is below the threshold during a small gap which has been annotated in the ground-truth. As a result, two ground truth fixations cannot be matched (two false-negatives) and one predicted fixation remains unmatched (one false-positive)

#### Event-level F1 score

Consecutive samples belonging to the same class can be aggregated into fixation or gap events, respectively. While simple to implement, the sample-based Cohen’s kappa does not assess well the level of agreement at this event-level. For example, if a single sample in the middle of a fixation is misclassified as a gap, this effectively breaks up the fixation event into two shorter fixations. While only one misclassified sample almost does not lower Cohen’s kappa score, this makes a large difference in the representation of events. Especially, if output events are later to be used in order to build perceptual metrics, such as measuring fixation duration as a proxy for overt visual attention, an accurate representation of detected fixations at the event-level is vital.

Because of this, a number of recent studies has suggested using an event-level F1 score for evaluation of similarity between two event sequences (Hooge et al., 2018; Startsev et al., 2019; Zemblys et al., 2019). This corresponds to a classical F1 score for event labels, after performing an event-matching procedure that matches overlapping events in the two sequences to each other. Since, in general, multiple events can overlap with each other, different matching criteria have been proposed in order to ensure a unique matching. For example, Hooge et al. (2018) assign the earliest overlapping event in the predicted sequence to a given ground-truth event, while Zemblys et al. (2019) match events with the largest overlap in time.

In our evaluations, we follow the recommendation by Startsev et al. (2019) to use an intersection-over-union (IoU) score, with a minimum criterion of 0.7, to match two events to each other. This recommendation follows from an analysis of different sample- and event-level metrics with respect to their ability to discern the output of event-detection algorithms from different random baseline models, including also event-level baselines.

Here, we evaluate event-level F1 scores only for fixations, not for gaps. It is important to realize that, by construction, the event-level F1 score penalizes different types of errors with distinct weights. In the simplest case, a single fixation, which cannot be matched to any ground truth event, is counted as one false-positive error (Fig. 3a). In contrast, if two fixations are predicted in place of one in a manner that the matching criterion is not fulfilled by either, e.g., because of fluctuations in gaze velocity surpassing the velocity threshold transiently, this is counted as two false-positive predictions and one false negative for the unmatched ground-truth fixation, i.e., as a three-fold error (Fig. 3b). Similarly, failing to detect a gap between two annotated ground-truth fixations can count as three errors (Fig. 3c). In summary, event-level F1 score particularly penalizes fragmentation and erroneously merged events.

**Fig. 4:**
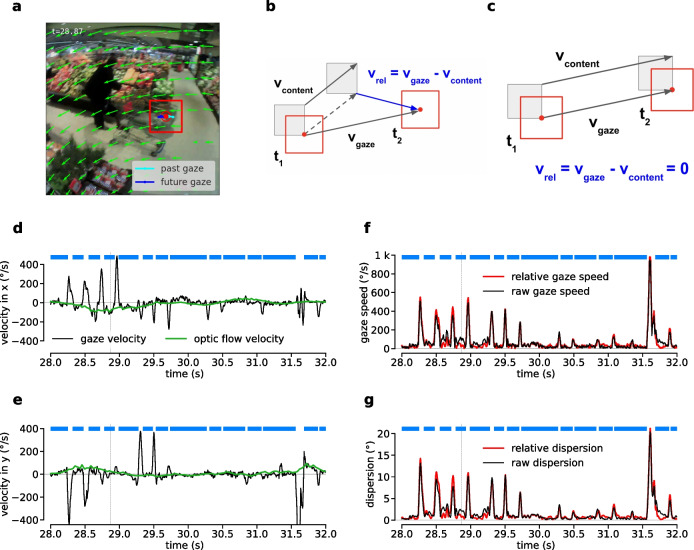
Optic flow compensation. **a** Snapshot of one frame during a fixation in a recording from the dynamic dataset. The subject is fixating the corner of a shopping cart while turning their head towards the right. **b** Illustration of optic flow compensation. From one frame at time $t_1$ to the next frame at time $t_2$, gaze moves with the gaze velocity $v_{gaze}$ to a new point, while the local visual content, which was previously co-located with the gaze point, moves to a different point with the velocity $v_{content}$. A relative gaze velocity with respect to the local visual content can be calculated by subtracting the two velocities: $v_{rel} = v_{gaze} - v_{content}$. Image content velocity can be estimated by optic flow estimation algorithms. **c** In the case that the observer is fixating, gaze velocity and the velocity of local image content approximately match and so, relative gaze velocity is close to zero, $v_{rel} \approx 0$. **d** Example traces for horizontal components of raw gaze velocity and optic flow velocity. The time point of the snapshot is marked by a *vertical dashed line*. Fixations are marked in *blue* at the top. Global optic flow velocity (*green*) almost seems like a baseline to the raw gaze velocity (*black*). **e** Same as (d) for the vertical components. **f** Same as (d), but showing the absolute values of the raw velocity vectors, respectively. Optic flow compensation leads to lower relative gaze speeds during fixations. **g** Same as (f), but showing gaze dispersion and relative gaze dispersion

### Fixation-detection algorithms

#### Standard I-VT and I-DT algorithm

We base our implementation of the standard I-VT algorithm on the work by Salvucci & Goldberg (2000). More concretely, we start by independently smoothing the raw *x*- and *y*-component of the gaze signal using a Savitzky–Golay filter with a polynomial of 3rd order and a window length of 55 ms. Then, gaze velocity in scene camera space is calculated from the forward differences of the position signal. A velocity threshold $v_{thr}$ is then applied by calculating gaze speed as the length of the velocity vector and classifying all samples with a speed below the threshold as belonging to a fixation, while all other samples are counted as gap samples. Velocities were technically calculated in units of *pixels per second*, and velocity thresholds implemented in the same way, however, for presentation, we converted values to *degrees of visual angle per second* ($^{\circ }\!/\text {s}$) according to the resolution and field of view of the scene camera for better understanding.

Similarly, we also built a version of the standard I-DT algorithm (Salvucci & Goldberg, 2000). To quantify dispersion, we use its previous definition as the sum of differences between the maximum and minimum *x*- and *y*-coordinates of gaze position, respectively, within a moving time window of 25 ms length centered about current time. We then apply a dispersion threshold, $d_{thr}$, to perform classification into fixation (dispersion is smaller than $d_{thr}$) and gap samples (dispersion is larger than $d_{thr}$).

In the following, whenever referring to the I-VT and I-DT algorithm at the same time, we will denote this by I-XT algorithm.

#### Event-based post-processing

The basic I-XT algorithm derives predictions for each gaze sample individually, i.e., does not take temporal correlations into account. Therefore, after converting sample-wise predictions by the I-XT algorithm into a time series of events, an event-based post-processing stage is employed.

First, a *microsaccade filter* is applied, removing gaps whenever their amplitude is below a given threshold value, $a_{thr}$, referred to as the minimum saccade amplitude, or if they are shorter than a given time threshold, $t_{thr}$, referred to as the minimum saccade length. Here, gap amplitude is calculated as the angular distance between the start and end gaze points of the gap. When a gap event is removed, neighboring fixation events are merged.

Second, in order to remove fragments too short to correspond to physiological fixations, a *short-fixations filter* is used for removing all fixations which are shorter than a given minimum duration, $d_{min}$.

We refer to the I-XT algorithm together with this event-based filtering stage as I-XT+F algorithm. It has been described, e.g., in Komogortsev et al. (2010); Olsen (2012); Hooge et al. (2022).

#### Strategy 1: Head-motion compensation

As pointed out earlier, applying the I-XT algorithm naively to head-mounted eye-tracking data often leads to erroneous outputs, since stabilization reflexes, such as VOR, can generate head-relative gaze velocities comparable to those of saccades (Sparks, 2002) (cf. also Fig. 2c,d). To address this problem, we propose a head-motion compensation as a pre-processing stage to the I-XT algorithm. In the following, we will deal with the I-VT and I-DT algorithm separately.

Consider the case of a fixation during head-turning, i.e., a case of VOR-based gaze stabilization (e.g., at a time of 28.8 s in Fig. 4a, Suppl. Video 2). Assuming perfect retinal stabilization of visual content, any change in gaze position needs to be equal in direction and magnitude to the corresponding head-relative displacement of the visual target. In terms of the recorded scene camera image, this means that the estimated gaze point needs to move in unison with the image content surrounding it. More formally, let $v_\textrm{gaze}$ denote the velocity of the gaze point and $v_\textrm{content}$ a suitably defined velocity of the surrounding image content. Note, both are measured in the scene-camera image.

Then

$$ v_\textrm{rel} = v_\textrm{gaze} - v_\textrm{content} $$

corresponds to the component of gaze displacement that is not consistent with the concurrent displacement of image content. In the following, we refer to $v_\textrm{rel}$ as *relative gaze velocity*. Since, assuming a perfect gaze estimator, local image content surrounding the gaze point corresponds to the visual content projected onto the retina, retinal stabilization and therefore fixating is equivalent to

$$ v_\textrm{rel}\approx 0. $$

Similarly, relative gaze velocity being far from zero is equivalent to a shift in image content projected onto the retina and thus falls under our functional definition of a gap (Fig. 4b). In other words, expressing gaze velocity relative to a coordinate system co-moving with the local image content renders the assumptions of the basic I-VT algorithm to conform with a functional definition of the term fixation.

One way to obtain the vector $v_\textrm{content}$ is to use an optic flow estimation algorithm. Both dense and sparse approaches to optical flow calculation are available (Bouguet et al., 2001; Farnebäck, 2003), which calculate velocities for each pixel or a grid of pixels, respectively. Ideally, $v_\textrm{content}$ would reflect the average velocity of an image patch corresponding to the foveal field of view, i.e., only a few degrees in diameter and centered around the gaze point. In practice, however, this is challenging to achieve, since the position of the estimated gaze point is subject to error. Confounding factors such as changing lighting conditions, headset slippage, and the unknown distance of the gaze target can lead to angular errors up to $5^{\circ }$ in visual space (Tonsen et al., 2020). Measurement noise in the position of the gaze point in turn leads to noise in the estimation of local optic flow. One way to alleviate this issue could be averaging optic flow vectors in a larger area around the gaze point. We tested this idea, using averaging windows of various sizes, but with regard to fixation-detection performance found no difference to using a simple global average of optic flow vectors (data not shown). Therefore, we decided to use global optic flow in our final implementation. However, this is likely dataset-dependent. We hypothesize that using local optic flow would be more advantageous for a dataset with more and large enough external objects moving independently from the observer.

Since global optic flow is tightly coupled to head motion, our approach effectively accounts for gaze stabilization movements, which are in place to compensate for head motion, i.e., VOR or OKN. In our implementation, global optic flow is computed by averaging optic flow vectors over a grid of 11$\times$11 equally spaced points which are calculated using the Lucas–Kanade method (Bouguet et al., 2001). Finally, relative gaze velocity is then used as an input to a standard I-VT algorithm.

Since head motion is one of the main confounding factors for fixation detection in head-mounted eye tracking, relative gaze velocity exhibits a stronger correlation with fixations in our dataset than raw gaze velocity (Fig. 4d-f). However, it is important to note that smooth pursuit movements, which are included in our functional fixation definition, are not accounted for by our head-motion compensation stage.

We now turn to the I-DT algorithm. Similar to the above construction, we aim at defining a *relative dispersion*, $d_\textrm{rel}$, which is close to zero for fixation samples, and larger for gaps. Note, in head-mounted eye trackers, the raw gaze position does not fulfill this criterion for gaze-stabilization reflexes like VOR. In this case, the gaze point is actually co-moving with the image from the head-fixed scene camera. Therefore, for each time point, we calculate a relative gaze trajectory, representing deviations from an ideal stabilized gaze trajectory, the latter corresponding to the gaze point moving in unison with the current image content. This ideal stabilized gaze trajectory is obtained by integrating optic flow vectors from the current time on. Relative dispersion is then calculated from the deviation of the actual gaze trajectory from this ideal trajectory.

More specifically, in our implementation, we calculate relative dispersion within a moving time window of 25 ms around the central frame. Starting from the central frame, we integrate global optic flow vectors backward and forward in time for half of the length of the time window, respectively, and add this to the current gaze position. This yields the absolute coordinates of an ideal trajectory, which gaze would follow in the case of perfect gaze stabilization w.r.t. global image shifts. Subtracting this from the actual gaze estimates in that window gives the component of the gaze trajectory that is not consistent with the global optic flow in the scene. Dispersion is then calculated from the resulting time series according to the original definition (Salvucci & Goldberg, 2000) and in the following is referred to as *relative dispersion* (Fig. 4g). Note, that for fixations with or without concurrent head movements, by construction, relative dispersion fulfills the criterion of $d_\textrm{rel} \approx 0$, and can thus be used as input to a standard I-DT algorithm.

In the following, whenever relative velocity or dispersion is used as input to a fixation-detection algorithm, we indicate this by a trailing superscript “rel” (as, e.g., in I-VT$^\textrm{rel}$).

#### Strategy 2: Adaptive threshold

Furthermore, we built a modified version of our head-motion compensated I-XT algorithm, which includes a linearly adaptive threshold. This is motivated by the observation that even after optic-flow-based head-motion compensation, gaze velocity tends to fluctuate stronger during periods of intense head motion than during periods of no head motion. One reason for this could be that high-velocity head motion often leads to image blur of the scene camera image, degrading the performance of optic flow algorithms, and thus the accuracy of head-motion compensation. Another factor might be fluctuations in the gaze-estimation algorithm, which is based on eye images, due to changing lighting conditions and potential headset slippage during the movement.

Therefore, we hypothesized that having a threshold-based algorithm, but with an adaptive threshold that depends on the intensity of head motion, could increase the overall performance of the algorithm.

To test this idea, we developed an I-XT algorithm with an internal threshold, which depends linearly on the average magnitude of optic flow in a time window, the latter being a proxy for the intensity of head motion. We call this adaptive algorithm the I-VAT or I-DAT, respectively, in the following. Algorithmically, we modeled the adaptive threshold according to the function

$$T(t) = T_0 + g\cdot o_{RMS}(t)$$

where $T_0$ is a baseline minimum threshold value, *g* is a gain factor, and $o_{RMS}$ is the length of the vector containing the component-wise root mean squared optic-flow velocity in a time window around the current time point. The length of this window is another free parameter of the algorithm.

#### Strategy 3: Coherent tuning

As mentioned earlier (see Related work), little attention is given in the literature to the choice of post-processing parameters. Often, they are set according to estimates of physiological parameters, but varying among studies (Hooge et al., 2022). A recent study, however, reported that the choice of post-processing parameters is crucial to modifying the output of a sample-based event-classification stage (Hooge et al., 2022). Specifically, they find that for data with moderate quality or higher, the choice of post-processing parameters can play an even more significant role than the choice of the classifier algorithm.

Here, we propose to include the parameters of the event-based post-processing stage in a global optimization of all parameters of a given algorithm with respect to an appropriate performance metric. In our experiments, we optimize event-level F1 score for each algorithm. As we will show, the optimal post-processing parameter set can depend on the parameters of the sample-based classifier stage. Therefore, we performed a global optimization for all algorithms presented. Generally, optimization is achieved by performing a grid-search over pre-defined ranges of plausible parameter values, maximizing event-level F1 score with respect to fixations on our annotated dataset, if not stated otherwise (see Suppl. Table 1).

#### Alternative algorithms

Here, we will briefly comment on our implementation of already published algorithms for fixation detection, which we included in our evaluation.

First, we used the REMoDNaV algorithm (“Robust Eye Movement Detection for Natural Viewing”) (Dar et al., 2020). While REMoDNaV was originally developed for remote eye tracking, it was specifically tailored to enable more robust fixation detection for dynamic video stimuli. This robustness is achieved by using an adaptive velocity threshold (very similar to Nyström & Holmqvist, 2010), which depends on an estimate of the signal-to-noise ratio in the data. In short, REMoDNaV first chunks the signal into compartments separated by major saccades, which are detected using an initial velocity threshold. After that, for each segment, minor saccades are detected using an iterative algorithm which adaptively lowers the velocity threshold until it converges at a safety margin above the estimated level of noise in the data. Furthermore, REMoDNaV uses additional heuristics to enable detection of smooth pursuit events and of post-saccadic oscillations.

We based our implementation of REMoDNaV on the author’s implementation^1^. However, to enable a quantitative comparison with our evaluation metrics, we chose to re-label detected smooth pursuit events as functional fixations. All other types of detected events do not play a role in our evaluation scheme since we evaluate event-level F1 scores only for fixations. To establish a fair comparison, we also performed a parameter optimization for the REMoDNaV algorithm. Since the algorithm has 17 internal parameters, we simplified this task, first, by assuming default values for all parameters which pertain to detection of post-saccadic oscillations. Second, we coupled corresponding parameters for fixation detection and smooth pursuit detection to the same values. Third, we identified parameters which, after setting plausible initial values, would not alter detection performance on our dataset in explorative experiments (e.g., the *maximum initial saccade frequency* (Dar et al., 2020)).

This way we identified a subset of seven most relevant parameters, including pre- and post-processing parameters (i.e., *noise factor*, *start velocity threshold*, *minimum fixation duration*, *maximum initial saccade frequency*, *size of saccade context window*, *size of Savitzky–Golay filter*, *smoothing median-filter size*). Finally, we exhaustively grid-searched this seven-dimensional parameter subspace optimizing for event-level F1 score (see Suppl. Table 1).

In addition, we also implemented a modified algorithm REMoDNaV$^{rel}$ featuring a head-motion compensation stage, similar to ours. For this, we modified the calculation of gaze velocity in the code, to yield a *relative* gaze velocity, using global optic-flow, in an analogous way to above.

As a second baseline, we used the algorithm by Steil et al. (2018), which was developed specifically for head-mounted eye trackers. As explained above (see Related work), this algorithm is based on calculating the visual similarity between gaze-centered patches from consecutive frames of the scene camera. Fixation detection is then performed by thresholding a time series of visual similarity scores. We used the same patch-similarity network as in the original study (Zagoruyko & Komodakis, 2015). For post-processing, we used the same heuristics as in our I-XT algorithm. To account for potential variations in the field of view of our scene camera with the one used in the original study, we varied the side length of the cropped image patches between 50 and 300 pixels. In total, our implementation is then governed by five parameters: the crop size, a similarity threshold, and three post-processing parameters. We also optimized this parameter set with respect to event-level F1 score on our dataset.

As a third alternative, we initially considered the algorithm proposed by Kinsman et al. (2012). Note, our velocity-based algorithm with head-motion compensation and optimized post-processing filters, i.e., I-VT$^\textrm{rel}$+F, is conceptually similar. Our approach, however, differs in that we use an additional micro-saccade filter, not only a filter for short fixations, and in the coherent optimization of all parameters, including post-processing stages. It therefore represents a related, but enhanced approach, putting Kinsman et al. (2012) at an unfair disadvantage. We therefore refrained from including it in the reported comparison.

## Results

### Analysis of the I-VT+F algorithm

In this section, we first discuss some general observations with respect to the proposed strategies in conjunction with the I-VT+F algorithm. Note, the following results represent optimized performance scores, i.e., even if one algorithm parameter is fixed at a given value, the other parameters have been optimized for optimal algorithm performance w.r.t. event-level F1 score. All reported performance scores are five-fold cross-validated with per-recording results averaged for each fold.

**Fig. 5:**
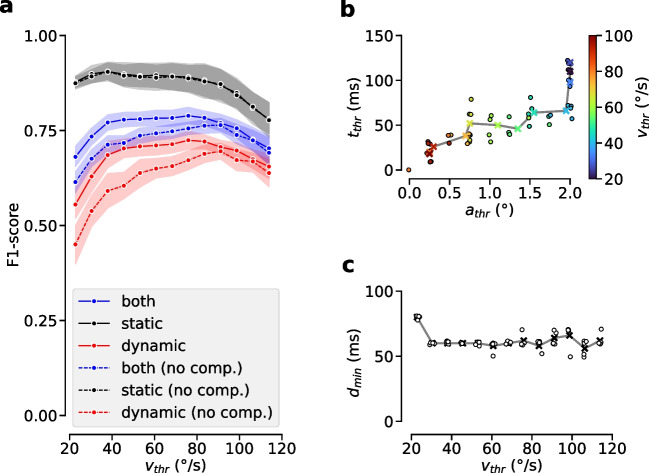
Optimization of the I-VT algorithm. **a** F1-score in dependence of the internal velocity threshold value $v_{thr}$, for dynamic (*red*), static (*black*) and both datasets (*blue*). *Solid lines* show performance with head-motion compensation, *dashed lines* without. The performance optimum for the dynamic dataset is flatter when applying head-motion compensation, while static performance remains high. *Error bars* show standard deviation over folds. **b** Optimal internal parameters of the micro-saccade filter in dependence of the velocity threshold value (*color-coded*). Individual points show optimum values for each of the five folds, the *grey line* connects the mean values for each fold. Lower velocity thresholds require higher values for $a_{thr}$ and $t_{thr}$. **c** Optimal minimum fixation duration $d_{\min }$ of the short-fixation filter in dependence of the velocity threshold. *Points* show individual results for each fold, as in (b). The optimal minimum fixation duration is largely independent from the velocity threshold with $d_{\min } \approx 60 \,{ms}$

#### The effect of head-motion compensation

It is our goal to devise an algorithm for fixation detection in head-mounted eye tracking that – after being tuned to the employed eye tracker – for most use cases can be applied out-of-the-box. Considering the differences between the static and dynamic scenario, however, the question arises whether a single choice of algorithm parameters can actually yield optimal performance in both conditions.

To investigate this question, we first tuned the algorithm parameters of the plain I-VT+F algorithm, i.e., without head-motion compensation and with a non-adaptive velocity threshold $v_{thr}$, on the static and dynamic dataset separately. Indeed, we observed a difference between the resulting optimal velocity threshold values, which is around $90^{\circ }\!/\text {s}$ for the dynamic dataset, but only around $40^{\circ }\!/\text {s}$ for the static one. These optima can also be appreciated when plotting tuning curves for each condition, i.e., the F1 score of the optimized algorithm as a function of the velocity threshold (dashed lines in Fig. 5a). Note, the higher optimal value for the velocity threshold in the dynamic setting is in line with previous findings for head-mounted eye tracking (Steil et al., 2018).

Since the occurrence of VOR-based gaze stabilization is a salient difference between the dynamic and static condition, we hypothesized that our head-motion compensation stage could help to diminish the observed tuning differences. Indeed, when compensating for head motion, i.e., using the I-VT+F$^\textrm{rel}$ algorithm, we found that tuning curves changed appreciably for the dynamic dataset (red solid line in Fig. 5a). First, the optimum shifted down to approximately $75^{\circ }\!/\text {s}$. Second, and most importantly, this was accompanied by a general increase in F1 over the whole range of tested velocity thresholds, in effect flattening out the whole tuning curve. This shows that head-motion compensation renders the algorithm more robust with respect to the exact choice of velocity threshold. In particular, with head-motion compensation lower than optimal velocity thresholds (between 50 and $80^{\circ }\!/\text {s}$) could be used for the dynamic dataset without considerably compromising algorithm performance. As expected, the tuning curve for the static condition did not change significantly, since head motion only rarely occurred in this scenario.

As a consequence, when tuning I-VT+F$^\textrm{rel}$ on both datasets simultaneously (blue solid line in Fig. 5a), the resulting optimal velocity threshold ensured a close to optimal F1 score for both the static and dynamic dataset individually. Overall, these findings demonstrate that only after head-motion compensation, a single set of algorithm parameters can be found, which renders fixation detection close to optimal in both the dynamic and static regime.

**Fig. 6:**
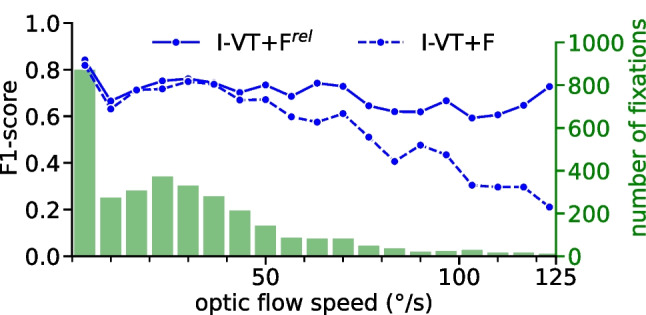
Fixation-detection performance depending on optic flow speed. F1 scores for I-VT+F$^\textrm{rel}$ (*blue solid line*) and I-VT+F (*blue dashed line*) algorithm, when evaluated and binned by maximum speed of optic flow for each detected fixation. In the limit of no optic flow, when the observer’s head is approximately stationary, both algorithms converge to the same performance. For optic flow speeds $> 50^{\circ }\!/\text {s}$, F1 score for I-VT+F monotonically decreases with increasing speeds. For I-VT+F$^\textrm{rel}$, however, the F1 score stays approximately constant, attesting to the effectiveness of the head-motion compensation stage in the presence of faster head movements

A complementary way of showing this is by grouping fixations across both datasets by concurrent optic-flow speed and measuring average fixation-detection performance for each group separately (Fig. 6). This analysis reveals that the performance of I-VT+F indeed decays with increasing optic-flow speed, while I-VT+F$^\textrm{rel}$ retains high F1 scores even in the presence of strong head movements. In the limit of no head motion, both algorithms deliver the same performance as expected.

#### Global optimization

Next, we asked whether a tuning of the algorithm parameters needs to be performed globally (as above), or whether one can separate the tuning of the sample-based classifier from the tuning of the post-processing filters. In particular, splitting the tuning process would be computationally more efficient.

We investigated this question by optimizing the post-processing parameters on the whole dataset for decreasing values of the velocity threshold $v_{thr}$ (Fig. 5b,c). We found that optimal values for both minimum saccade amplitude $a_{thr}$ and minimum saccade duration $t_{thr}$ increased with decreasing $v_{thr}$ (Fig. 5b). Indeed, the optimal value for $a_{thr}$ gradually changed from 0.25 to $2.00^{\circ }$, when varying $v_{thr}$ from over $100^{\circ }\!/\text {s}$ to around $20^{\circ }\!/\text {s}$. Similarly, the optimal value for $t_{thr}$ gradually changed from 25 to 150 ms.

One plausible interpretation of this is that lowering the velocity threshold towards the noise level of the gaze velocity signal leads to increased fragmentation and more false saccade detections. To recover event-based F1 scores, the micro-saccade filter therefore needs to remove those falsely detected gaps from the event sequence. Indeed, since $a_{thr}$ and $t_{thr}$ represent the upper bounds of the selection-criteria for gap removal, increasing these values results in the deletion of more gap events from the sequence.

For the minimum fixation duration parameter $d_{min}$ of the short-fixation filter, in contrast, we found no dependency on the velocity threshold $v_{thr}$ (Fig. 5c). Removing fixations shorter than around 60 ms was optimal for all configurations tested. This value corresponds well to previous recommendations in the literature (Hooge et al., 2018).

**Table 1:** Comparison with other algorithms

	*F*1	$F1_{static}$	$F1_{dynamic}$	$\kappa _{samples}$
I-VAT+F$^{rel}$	0.80 (0.04)	0.90 (0.06)	0.74 (0.04)	0.76 (0.04)
I-DAT+F$^{rel}$	0.80 (0.05)	0.89 (0.07)	0.74 (0.05)	0.77 (0.03)
I-VT+F$^{rel}$	0.78 (0.05)	0.89 (0.08)	0.71 (0.05)	0.76 (0.03)
I-DT+F$^{rel}$	0.77 (0.06)	0.89 (0.09)	0.70 (0.06)	0.75 (0.04)
I-VT+F	0.76 (0.05)	0.87 (0.09)	0.68 (0.07)	0.72 (0.04)
I-DT+F	0.73 (0.05)	0.87 (0.10)	0.65 (0.06)	0.71 (0.05)
I-DT	0.68 (0.05)	0.85 (0.10)	0.57 (0.08)	0.76 (0.03)
I-VT	0.66 (0.04)	0.86 (0.08)	0.53 (0.08)	0.74 (0.03)
ReMoDNaV$^{rel}$	0.65 (0.05)	0.68 (0.10)	0.62 (0.07)	0.65 (0.04)
ReMoDNaV	0.59 (0.05)	0.65 (0.14)	0.56 (0.06)	0.64 (0.05)
Steil et al.+F	0.58 (0.07)	0.67 (0.11)	0.52 (0.06)	0.44 (0.10)
Steil et al.$^{original}$	0.56 (0.06)	0.66 (0.11)	0.50 (0.05)	0.33 (0.11)
ReMoDNaV$^{dynamic}$	0.55 (0.08)	0.48 (0.16)	0.60 (0.06)	0.65 (0.04)
ReMoDNaV$^{static}$	0.50 (0.08)	0.85 (0.05)	0.27 (0.16)	0.61 (0.06)

5-fold cross-validated evaluation scores for each algorithm tested. Overall event-level F1-score was optimized. Standard deviation over folds is given in round brackets

First column: overall event-level F1 score on all data; second column: F1 score on only the static subset; third column: F1 score on the dynamic subset; fourth column: sample-level Cohen’s kappa on all data

**Fig. 7:**
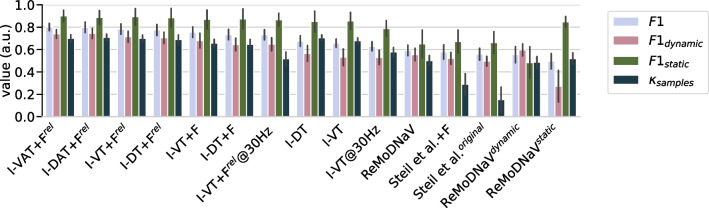
Comparison with other algorithms. Mean event-level F1-scores for each algorithm tested (same data as in Table 1). Overall F1-score (*light blue bars*) was optimized. *Pink and green bars* show validation F1-scores on only the dynamic or static dataset, respectively. *Dark blue bars* show evaluation scores using the Cohen’s $\kappa$ metric at sample-level

In summary, our findings show that when maximizing event-level F1 scores, the parameters of the sample-based classifier and the parameters of event-based post-processing stages cannot be assumed to be independent. In particular, the velocity threshold and the parameters of the micro-saccade filter are entangled with each other. Therefore we conclude that a joint tuning of all parameters is crucial for optimal algorithm performance in terms of event-level F1 score. Consequently, we always performed a global optimization of all parameters for each algorithm tested in the following (see Suppl. Table 1).

### Strategies for improving threshold-based fixation detection

We now turn to a performance evaluation of our suggested strategies for improving threshold-based algorithms in the context of head-mounted eye tracking. For this, we quantified event-level F1 scores for algorithm variants with different ablations from the full combination of all strategies suggested, i.e., (i) adaptive thresholding, (ii) head-motion compensation, and (iii) optimized post-processing filters (Table 1, Fig. 7).

**Fig. 8:**
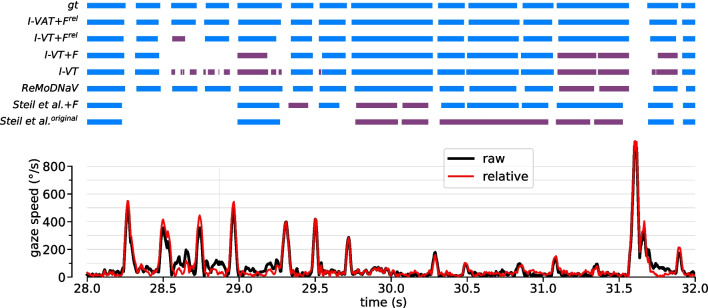
Example predictions from dynamic dataset. *Top:* Ground-truth annotations (gt) and predicted events for a selection of different algorithms. Correctly predicted fixations are highlighted in *blue*. Predicted fixations which cannot be matched to the ground-truth (false positives) are colored in *purple*. Note that false negatives, i.e., failing to predict an annotated ground truth fixation, are not highlighted in color in this visualization, but are visible only implicitly, e.g., in some cases, through a larger “white” gap in the predicted sequence. *Bottom:* Raw (*black*) and relative (*red*) angular gaze speed. The observer is moving their head rapidly to the right at t $\approx$ 28.8 s (see Suppl. Video 2 and Fig. 1). Without post-processing filters and without optic flow compensation, the basic I-VT algorithm produces quite fragmented predictions here, since the velocity threshold value (in this case 91 ${^\circ }$/s) lies in the range of speed fluctuations due to head motion. Post-processing (I-VT+F) removes a lot of these fragments, but leaves a long gap instead, missing some of the annotated ground-truth fixations during this period. Adding optic flow compensation (I-VT+F$^\textrm{rel}$) enables the algorithm to detect these fixations during head motion

**Fig. 9:**
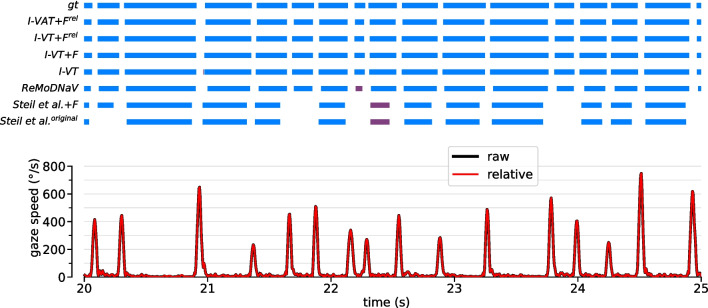
Example predictions from static dataset. Same as in Fig. 8, but using an example from the static dataset. Since there is almost no head motion in this case, many of the top algorithms yield very similar predictions

#### Performance gains through adaptive thresholding

Overall, the best event-level F1 scores on the whole dataset were achieved by a combination of all strategies, i.e., by our algorithm variants I-VAT+F$^\textrm{rel}$ and I-DAT+F$^\textrm{rel}$ (Table 1). Both achieved an F1 score of 0.80. This shows that both velocity and dispersion are equally useful features for fixation detection, which is not surprising considering their high correlatedness (see Fig. 4f,g). Both algorithms also reached the respective maximum F1 scores when evaluated on only the static (0.90) or dynamic dataset (0.74). When inspecting example output sequences of the velocity-based algorithm, we indeed found a high level of agreement between predicted and ground-truth event sequences for both conditions (dynamic example: Fig. 8; static example: Fig. 9). The two algorithms also performed best with respect to the sample-based Cohen’s kappa metric $\kappa _{samples}$. Overall, however, $\kappa _{samples}$ was found to correlate only weakly with event-level F1 score (cf. Table 1).

We asked, what was the specific contribution of having an adaptive threshold versus a standard fixed threshold implementation of the algorithm (i.e., comparing with I-VT+F$^\textrm{rel}$ and I-DT+F$^\textrm{rel}$). While removal of the adaptive threshold led to an overall performance drop of 2–3% in event-level F1 on the whole dataset, the effect was more pronounced on the dynamic dataset with a performance loss of 3–4% and only around 1% for the static dataset. This difference is intuitive, since adaptive thresholding is governed by head motion intensity in our algorithm.

Having an adaptive velocity threshold enables I-VAT+F$^\textrm{rel}$ to perform robust fixation detection even for fixations during rapid head motion. This can be seen in the example at around a time of 28.8 seconds (Fig. 8 and Suppl. Video 2). A more in-depth analysis showed that the adaptive velocity threshold, in effect, varied between values of 47 $^{\circ }$/s up to around $300^{\circ }\!/\text {s}$ on our dataset, depending on the current estimate of head-motion intensity. This shows that the algorithm is able to perform fine-grained fixation detection in more static situations, while ramping up the velocity threshold during times of strong head-motion of the subject suppressing noisy and fragmented output predictions. Similarly, for the corresponding dispersion-based algorithm I-DAT+F$^\textrm{rel}$, the adaptive dispersion threshold varied between a minimum value of 1 $^{\circ }$ and up to maximum values of approximately 10 $^{\circ }$ for dynamic recordings.

The average optimal parameter set of I-VAT+F$^\textrm{rel}$ corresponded to the following values: minimum velocity threshold $v_{0}=42$ $^{\circ }$/s (560 px/s), gain factor $g=0.70$, window size $w=205$ ms, and post-processing parameters $a_{thr}=1.7^\circ$, $t_{thr}=42$ ms, and $d_{\min }=54$ ms (see Methods for parameter definitions).

#### Performance gains through head-motion compensation

Next, we ablated the pre-processing head-motion compensation stage in order to precisely quantify its contribution in terms of event-level F1 score. As observed above for the tuning curves, we expect a stronger effect for the dynamic than for the static dataset.

Indeed, when going from I-VT+F$^\textrm{rel}$ to I-VT+F, overall performance dropped by 2%, which consisted of a drop of 3% on the dynamic dataset, but no performance loss on the static dataset. Similarly, for the dispersion-based algorithm, F1 decreased by 5% on the dynamic dataset, but only 2% on the static dataset, and 4% overall.

Interestingly, when inspecting our example sequence, we found that the plain I-VT+F algorithm completely failed to detect fixations during fast head-turns (time 28.8 s in Fig. 8). This shows how both adaptive thresholding and head-motion compensation work together to enable robust fixation detection in more dynamic settings.

Notably, the fixed velocity threshold value for this algorithm variant was 68 ${^\circ }$/s on average, so somewhat higher than the minimum velocity threshold for the adaptive threshold algorithm. One plausible interpretation of this is that since this velocity threshold must be applied to all settings at all times, using a higher velocity threshold is necessary to avoid fragmentation of predicted events in dynamic situations. These findings are well in line with our observations described above for the tuning curves.

### Performance gains through post-processing

Next, we wanted to gauge the effect of the event-based post-processing filters on algorithm performance. Again, we performed ablation experiments removing the complete post-processing stage and optimizing effectively only the velocity threshold as the only parameter left for I-VT, or the dispersion threshold for I-DT, respectively.

This manipulation led to a 10% overall performance loss for the velocity-based algorithm, and 5% for the dispersion-based algorithm, respectively. Again, the effect was much stronger on the dynamic than on the static dataset. For I-VT, dynamic F1 decreased by 15%, while static F1 dropped only by 1%. Similarly, for I-DT, the respective performance losses amounted to 8% and 2%, respectively.

These numbers can be explained by a drastic increase in fragmentation without the post-processing filters in place to remove such fragments, especially during VOR-based gaze stabilization (see I-VT and Fig. 8). In the static case, event predictions still faithfully represent the ground-truth event sequence (see I-VT in Fig. 9). Interestingly, the fitted velocity-threshold in this case is with 91 ${^\circ }$/s on average even higher than in the analysis above. Presumably, with no post-processing in place, this avoids as much fragmentation as possible without compromising the basic functionality of the algorithm.

Overall, our findings show that event-based post-processing is of utmost importance for both velocity- and dispersion-based algorithms for removing artifacts from noise and other fluctuations in the input signal.

### Comparison with other approaches

#### ReMoDNaV

As a first alternative method, we evaluated the fixation-detection algorithm ReMoDNaV (Dar et al., 2020), which has been originally developed for remote eye-tracking data. At its core, ReMoDNaV utilizes an adaptive velocity threshold and has been optimized for the task of noise-robust fixation detection with dynamic stimuli such as video sequences (see Methods).

Despite having more parameters available for tuning, surprisingly, we found ReMoDNaV with an event-level F1 of 0.59 to perform significantly worse than I-XT algorithms on our dataset (Table 1). We hypothesized that this low performance score was reflective of a compromise resulting from the attempt to fit a fixed parameter set to both the static and dynamic dataset at the same time. Therefore, we fitted ReMoDNaV to both datasets separately.

Indeed, when tuned and evaluated on only one subset of the data, the algorithm performed comparably or slightly better than a plain I-XT algorithm. Event-level F1 on the static dataset was 0.85, when tuned on only the static dataset (see ReMoDNaV$^{static}$ in Table 1), which is 20% better than when tuned on both datasets together. F1 score on the dynamic dataset was 0.60, when tuned on dynamic data only (see ReMoDNaV$^{dynamic}$ in Table 1), which is slightly better than the performance of vanilla I-XT algorithms on the same data.

These findings indicate that the algorithm is overfitting to the respective tuning dataset, and suggest that the optimal parameter sets for static and dynamic input data are very distinct from each other. Indeed, when inspecting the two optima, we found a marked differences in the algorithm parameter *noise factor*, which determines the noise sensitivity of the adaptive velocity threshold.

We conclude that while ReMoDNaV was developed to deal with data of variable input quality and for dynamic stimuli in remote eye-tracking settings, it cannot account for the different input signal statistics encountered in head-mounted eye-tracking data. The adaptive velocity threshold in ReMoDNaV differs in so far from the one in our adaptive threshold algorithm I-VAT+F$^\textrm{rel}$, that it is set according to temporally varying statistics of the gaze signal itself, such as median value and mean absolute deviation of gaze velocity. In contrast, our adaptive threshold algorithms modulate the velocity or dispersion threshold, respectively, in relation to the intensity of optic flow in the scene camera, which is independent of gaze. In addition, ReMoDNaV filters out micro-saccades based on only temporal criteria, i.e., saccade and inter-saccade duration, but not by spatial amplitude, as in our algorithm.

#### REMoDNaV$^{rel}$

When equipping the ReMoDNaV algorithm with a head-motion compensation stage, the algorithm performed better on both data subsets than the vanilla implementation. Specifically, the performance on the dynamic dataset was even higher (0.62 event-level F1) than for ReMoDNaV$^{dynamic}$ (0.60), which was intentionally overfitted to the dynamic data. This shows, for a different algorithm, the usefulness of such head-motion compensation stage for fixation detection in head-mounted eye-tracking data. Overall, however, ReMoDNaV$^{rel}$ performance did not surpass the F1-score of simpler algorithms.

#### Fixation detection based on visual similarity

Next, we also evaluated the fixation-detection algorithm suggested by Steil et al. (2018), since this approach has been developed for head-mounted eye tracking specifically. It also represents a fundamentally different alternative to our approach, with fixation detection being based on the visual similarity of gaze targets and not on gaze dynamics (see Methods). In short, while algorithmically the method resembles an I-XT approach, the input signal in this case corresponds to a signal of visual similarity between consecutive gaze-centered image patches. Intervals of uninterrupted high visual similarity are classified as fixations.

Surprisingly, our re-implementation of the original method on our dataset performed significantly worse than the I-XT algorithm, with an event-level F1 score of only 0.56 (Table 1).

In order to enable a fairer comparison, we added several modifications to this algorithm. In the original publication, gaze patches were created by cropping rectangular regions of size 200x200 px around the gaze point. We hypothesized that when using a different camera as well as an entirely different dataset with differently sized objects in the view, a different crop size could be necessary. Therefore, we introduced the size of the cropped image patch as an additional algorithm parameter, varying between 50 and 300 pixels. Furthermore, we added a post-processing stage identical to our other threshold-based algorithms.

Using this improved algorithm (Steil et al.+F in Table 1), we found event-level F1 to increase only slightly by 2% from the original implementation. In addition, the F1 score was low for both static and dynamic dataset in comparison with most other algorithms.

When inspecting the algorithm output directly, we found that one of the main reasons for this was that the algorithm often failed to detect complete fixations in the presence of head motion (see Fig. 8). Despite its design based on visual similarity, in our hands the algorithm did not reliably identify such gaze-stabilization reflexes as fixations. This indicates that the calculated visual similarity scores might not be high enough during VOR-based gaze stabilization. We speculate that reasons for this could be image artifacts, e.g., image blur and/or noise in the prediction of the gaze point during head movements. Furthermore, there could also be a mismatch between the distribution of image patches from our recordings and the training dataset of the image similarity network used.

In addition, we observed that the algorithm also failed to predict some fixations in the static dataset (Fig. 9). By manual inspection, we found that this appears to happen especially when the visual content in the periphery of the gaze target is changing. Since our static dataset is mostly based on participants watching video stimuli (see Suppl. Video 1), this is a frequently occurring situation.

**Fig. 10:**
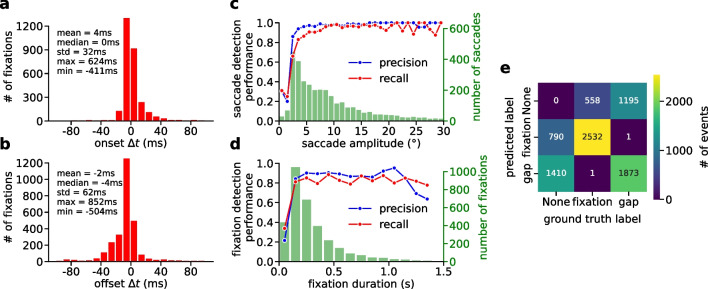
Other evaluation metrics. **a** Distribution of onset timing differences between ground-truth and predicted fixation events. Mean value, standard deviation, and maximum and minimum onset timing differences are indicated in the plot. **b** Same as in (a), but for timing differences in the offset of fixation events. **c** Precision (*blue*) and recall (*red*), when evaluated for “gap” events, interpreted as “saccades” in this plot, in dependence of the saccade amplitude. Most false predictions or missed saccades are in the region of small amplitudes. Since errors in saccade detection go together with errors in fixation detection, this means that most errors that the algorithm makes, are due to misdetection of saccades $<5^{\circ }$. *Green bars* show the distribution of saccade amplitudes in the dataset. Note that for this analysis, the IoU matching-threshold was set to 0 %, in order to detect the presence of predicted saccades, even if they were relatively short in comparison with their respective ground truth events. **d** Precision (*blue*) and recall (*red*) for fixations, in dependence of the fixation duration. Short fixations 100 ms are more likely to be not detected or falsely predicted. *Green bars* show the distribution of fixation durations in the dataset. **e** Confusion matrix of predicted against ground-truth events for the whole dataset. Matched events are along the diagonal of the matrix (using a minimum IoU matching-threshold of 0.7). Whenever an event cannot be matched to any event in the other sequence, it is matched to “None”, in this representation. Note that gaps are more difficult to match because of their very short duration. However, this does not affect F1 scores for fixations

#### Other evaluation metrics

The event-level F1 score is one way to measure similarity between a predicted event sequence and ground-truth annotations. However, in order to fully characterize the quality of a fixation detector, it is necessary to look at other evaluation metrics as well. Correspondingly, we performed a more thorough evaluation of our tuned best fixation detectorI-VAT+F$^\textrm{rel}$.

To provide another measure of agreement between two event sequences, Hooge et al. (2018) recommended quantifying the relative timing differences between the on- and offsets times of events, together with the standard deviation of these differences. Following this suggestion, we calculated timing differences between predicted fixations and annotated ground-truth events (Fig. 10 a, b). On average, we found a mean timing difference of 4 ms for the onset of annotated fixations, and -2 ms for the offset, indicating that there is effectively no systematic temporal misalignment of the predicted event sequence. The distribution of onset timing differences was slightly skewed towards positive values, with a standard deviation of 32 ms. For fixation offsets, we found a distribution slightly skewed towards earlier times, with a standard deviation of 62 ms. In extreme cases, timing was off by a few hundreds of milliseconds, which can happen, e.g., by a misdetection of a short fixation just before or after a relatively long fixation.

Next, in order to provide an error analysis of misdetections, we looked at the algorithm as a gap detector instead of a fixation detector. Since the output labels of the algorithm are binary, errors in the representation of fixation events are reflective of errors in predicting the neighboring gaps, and vice versa. We interpreted gaps as saccades and measured saccade-detection performance by means of appropriate event-level metrics, in this case event-level precision and recall.

When binning saccades according to their amplitude, we found that detection performance decreased drastically for saccade amplitudes smaller than $3\,^{\circ }- 4\,^{\circ }$ (Fig. 10c). This applies to event-level precision, i.e., predicted small saccades are much less likely to represent correct predictions, as well as to recall, i.e., small ground-truth saccades are more likely to be missed.

Since the distribution of ground truth saccades peaks at around $3^{\circ }$, this represents one major source of errors in the output of the algorithm in its current form. False prediction of small saccades leads to fragmentation of overlapping fixations, while failing to detect ground-truth saccades leads to erroneous merging of neighboring fixations.

We asked whether longer fixations would be more affected by these types of errors than shorter ones. To address this question, we performed a similar analysis, evaluating event-level metrics for fixations, binned by fixation duration (Fig. 10d). We found fixation-detection precision and recall to be low for fixations shorter than 100 ms, presumably due to some fragmentation in the output of the algorithm. Otherwise, we did not observe a strong dependency of precision or recall on fixation duration.

Since both, precision and recall, have relatively similar values, we conclude that the I-VAT+F$^\textrm{rel}$ algorithm is not dominated by one type of error, i.e., neither false predictions nor misdetections of fixations or saccades are over-represented among the remaining errors of the algorithm. This is also confirmed by the relative symmetry of the confusion matrix of the algorithm (Fig. 10e).

## Discussion

In this work, we explored strategies for enhancing standard thresholding algorithms for automated fixation detection in conjunction with head-mounted eye trackers.

To provide an experimental testbed, we curated a dataset of hand-labeled fixations for recordings obtained with the Pupil Invisible glasses by Pupil Labs. In particular, it comprises recordings both from dynamic and static scenarios, mimicking realistic use cases of this eye tracker. We publish the dataset along with this work.

Head motion being one of the main confounding factors for fixation detection in head-mounted eye tracking, we considered a head-motion compensation stage operating on the raw gaze signal. Utilizing estimates of global optic flow in the scene camera image, we transformed velocity and dispersion measures relative to a reference frame co-moving with scene camera image content. While by construction having little impact in static usage scenarios, we could confirm the positive effect of this pre-processing stage in dynamic settings.

Next, we explored adapting velocity or dispersion thresholds, respectively, relative to a proxy-measure of current head-motion intensity. Being motivated by increased gaze fluctuations during high-velocity head maneuvers, our analysis verified the benefit of this additional strategy of accounting for head motion.

Lastly, we demonstrated the positive impact on algorithm performance of a global optimization taking into account all pertinent algorithm parameters. Our results, in particular, attest to the dependency between parameters of pre- and post-processing stages, respectively, and thus highlight the importance of global, instead of, e.g., stage-wise tuning.

We analyzed the impact of all three strategies on event-level F1 scores and compared to results obtained with algorithms proposed previously. Overall, we found that a standard velocity-based thresholding algorithm in conjunction with head-motion compensation, an adaptive velocity threshold, and a global optimization of all algorithm parameters performed better than all alternative approaches. Our results therefore provide evidence that this algorithm class, while originally developed for remote eye trackers, when enhanced with the above strategies is well suited for providing reliable fixation detection in head-mounted eye tracking. In particular, by tuning on a diverse dataset, we obtain algorithm parameters that cover a wide range of use cases, comprising both highly dynamic as well as static scenarios. However, when dealing with a specific class of activity only, finetuning of algorithm parameters might improve performance even further. This might be particularly the case for activities not included in the dataset.

It is worth noting that optimal algorithm parameters generally also depend on the specific eye tracker used. For example, the optimal parameters of post-processing filters are likely influenced by the accuracy and precision of the employed gaze-estimation algorithm. While the optimal parameter set reported here is largely consistent with values found in the literature (e.g., see (Hooge et al., 2022) for post-processing parameters), re-tuning the parameters on a suitable dataset is likely necessary to ensure optimal performance for other eye trackers.

Our work also has a few limitations. By opting for a five-fold cross-validation scheme, care was taken to measure all reported performance metrics on recordings which were not part of the optimization itself. Our dataset, however, provides fixation labels which were generated by a single expert-annotator only. The agreement of distinct annotators in terms of event-level F1 score, on a different dataset, has previously been found to be approximately 0.93 (Hooge et al., 2018). For the sake of this discussion, we assume this finding to generalize to our dataset. It can then be viewed as a very rough estimate of an upper bound for the algorithmic detection of fixations. Note, the performance of our best performing algorithm (I-VAT+F$^\textrm{rel}$ on the static dataset) is indeed close to the inter-annotator F1 score reported in Hooge et al. (2018). That poses the question, as to whether our algorithm is truly close to being optimal in that sense, or whether it achieves such high F1 scores due to being tuned to the idiosyncrasies of the annotator of our dataset. In order to facilitate work on this question, we are publishing both the raw recordings as well as our fixation labels together with this work.

Even after head-motion compensation, a discrepancy remained between performance on the static and dynamic dataset, respectively. This is likely due to a number of reasons.

Hand-labeling fixation events which conform to the functional definition of gaze stabilization is more challenging for the dynamic than the static dataset. This is due to reduced precision of gaze estimates during intense head motion, direction-dependent gaze-estimation errors, and motion blur affecting camera images. This could have resulted in less consistent ground-truth labels in the dynamic dataset, implying an overall lower upper bound of achievable F1 score.

Our pre-processing approach relies on global optic flow to capture the effect of head motion. This, however, is only true for its dominant modes, namely pure rotations around the central body axis. These introduce a constant velocity field over the whole field of view and are thus robustly picked up by our method. Head tilts, however, generate non-constant, vertex-like velocity patterns, which are not well characterized by global averages of optic flow vectors.

More generally, optic flow estimation by means of the Lukas–Kanade algorithm becomes less accurate, e.g., when used on uniform image content (as e.g., in complete darkness), for large inter-frame camera rotations/displacement, and when image material is affected by motion blur. Head-motion compensation as implemented in this work, therefore cannot account for all changes in gaze-relevant image content.

Even after transforming gaze velocity or dispersion, respectively, smooth pursuits of objects, which are considerably smaller than the field of view, are not likely to be detected as fixations. The detection of smooth pursuits necessitates a local quantification of optic flow, essentially gauging the apparent velocity of the gaze-followed object. Since gaze-estimation errors of several degrees lead to a limited overlap of small gaze-followed objects with the estimated foveal field of view, such approaches are still out of reach.

We envision several routes for improving on the results reported here. To this end, note that progressively removing reasons hampering the hand-labelling of fixations, is also likely to improve automated fixation detection. With improved precision and accuracy of gaze estimates, derived velocity and dispersion time series will more reliably reflect true eye-in-head motion. In particular, increased precision (i.e., decreased gaze-independent jitter) will reduce the fragmentation of predicted event sequences. Increased accuracy will allow for more local estimation methods for quantifying the movement of image content. Scene cameras offering better image quality on the other hand, will allow for the more reliable estimation of optic flow, both locally and globally. We believe that these problems are likely to be addressed in the future through the further improvement of head-mounted eye-tracking hardware, which will in turn propel the development of ever more advanced algorithmic solutions to gaze estimation.

In order to solve the problem of separating fixational gaze shifts during head motion from saccades, our proposed algorithm for fixation detection effectively processes two independent data streams: the gaze signal and the scene camera video. In order to segment the gaze signal into more fine-grained event classes, the integration of data from additional sensors will most likely be necessary. For example, head and body movements can also be estimated from gyroscopic and inertial measurement unit (IMU) sensors routinely embedded in current head-mounted eye trackers. Through a comparison of local optic flow, global optic flow, and IMU data, VOR-based gaze stabilization potentially becomes distinguishable from smooth pursuits. More generally, we believe it will be advantageous to utilize a diverse set of complementary raw sensor and derived data streams as input for future fixation-detection algorithms.

While heuristic methods, as the ones discussed in this study, have the appeal of enabling insights into the specific role of each algorithm parameter, devising and tuning appropriate classification schemes quickly becomes unfeasible as the number of input variables increases. Appropriate data-driven methods in contrast can, when given enough training data, derive predictive decision criteria automatically. In conjunction with utilizing more input data streams, we therefore see a potential in using data-driven methods for gaze-event classification. Indeed, modern machine learning approaches have already been applied to the problem of fixation detection in remote eye tracking, albeit relying on measures of gaze dynamics only (Hessels et al., 2017; Zemblys, 2017; Zemblys et al., 2018; Hoppe & Bulling, 2016; Zemblys et al., 2019; Startsev et al., 2019). Such methods could be a functional replacement for the sample-based classification stage implemented in our study as a simple thresholding operation. As we have shown, introducing a more complex classifier by adaptively modulating the threshold height already led to an increase in fixation-detection performance. Therefore, sample-based classification could likely be further improved by using more expressive methods for this stage.

The importance of event-based post-processing stages has been highlighted before (Hooge et al., 2022) and becomes apparent also in our work. Being downstream of sample-wise classification, they effectively constitute a way of taking temporal correlations into account. Substituting the simple filters employed in this study by learnable post-processing stages, potentially realized in the form of recurrent or convolutional neural networks, would enable the algorithm to discover and take into account relevant event-level statistics more directly. Of particular interest in this regard are sequence-to-sequence machine learning approaches. While capitalizing on correlations present in input sequences, these models at the same time are explicitly designed to produce output sequences which faithfully reproduce the statistics of the target domain. The combination of using more sensors and input data streams as well as data-driven methods for event detection in head-mounted eye-tracking data are a promising line of future research.

## Supplementary Information

Below is the link to the electronic supplementary material.

Supplementary file 1 (mp4 1841 KB)

Supplementary file 2 (mp4 2274 KB)

Supplementary file 3 (mp4 12307 KB)

Supplementary file 4 (pdf 807 KB)

## Data Availability

The dataset generated during and analyzed for this study is available on https://osf.io/8en9v/.

## Footnotes

1 https://github.com/psychoinformatics-de/remodnav

## References

[CR1] Agtzidis, I., Startsev, M., & Dorr, M. (2019). 360-degree video gaze behaviour: A Ground-Truth data set and a classification algorithm for eye movements. In: Proceedings of the 27th ACM international conference on multimedia, MM ’19, pages 1007–1015, New York, USA, Oct. 2019. Association for Computing Machinery.

[CR2] Anantrasirichai, N., Gilchrist, I. D., & Bull, D. R. (2016). Fixation identification for low-sample-rate mobile eye trackers. In: 2016 IEEE International Conference on Image Processing (ICIP), pages 3126–3130.

[CR3] Andersson, R., Larsson, L., Holmqvist, K., Stridh, M., & Nyström, M. (2017). One algorithm to rule them all? An evaluation and discussion of ten eye movement event-detection algorithms. *Behavior Research Methods,**49*(2), 616–637. 27193160 10.3758/s13428-016-0738-9

[CR4] Baumann, C., & Dierkes, K. (2023). Neon Accuracy Test Report. *Pupil Labs*. 10.5281/zenodo.10420388

[CR5] Bouguet, J.-Y., et al. (2001). Pyramidal implementation of the affine lucas kanade feature tracker description of the algorithm. *Intel corporation,**5*(1–10), 4.

[CR6] Dar, A. H., Wagner, A. S., & Hanke, M. (2020). REMoDNaV: Robust eye movement detection for natural viewing. *Cold Spring Harbor Laboratory*, page 619254.

[CR7] David, E. J., Gutiérrez, J., Coutrot, A., Da Silva, M. P., & Callet, P. L. (2018). A dataset of head and eye movements for videos. In: Proceedings of the 9th ACM multimedia systems conference, MMSys ’18, pages 432–437, New York, USA, June 2018. Association for Computing Machinery.

[CR8] de Barbaro, K., Chiba, A., & Deák, G. O. (2011). Micro-analysis of infant looking in a naturalistic social setting: Insights from biologically based models of attention. *Developmental Science,**14*(5), 1150–1160. 21884330 10.1111/j.1467-7687.2011.01066.x

[CR9] Diaz, G., Cooper, J., Rothkopf, C., & Hayhoe, M. (2013). Saccades to future ball location reveal memory-based prediction in a virtual-reality interception task. *Journal of Vision,**13*(1), 20. 23325347 10.1167/13.1.20 PMC3587002

[CR10] Duchowski, A. T. (2002). A breadth-first survey of eye-tracking applications. *Behavior Research Methods, Instruments, & Computers,**34*(4), 455–470. 10.3758/bf03195475 12564550

[CR11] Engbert, R., & Mergenthaler, K. (2006). Microsaccades are triggered by low retinal image slip. *Proc. Natl. Acad. Sci. U. S. A.,**103*(18), 7192–7197. 16632611 10.1073/pnas.0509557103 PMC1459039

[CR12] Farnebäck, G. (2003). Two-Frame motion estimation based on polynomial expansion. *Image Analysis* (pp. 363–370). Berlin Heidelberg: Springer.

[CR13] Franchak, J. M., Kretch, K. S., Soska, K. C., & Adolph, K. E. (2011). Head-mounted eye tracking: A new method to describe infant looking. *Child Development,**82*(6), 1738–1750. 22023310 10.1111/j.1467-8624.2011.01670.x PMC3218200

[CR14] Hansen, D. W., & Ji, Q. (2010). In the eye of the beholder: A survey of models for eyes and gaze. *IEEE Transactions on Pattern Analysis and Machine Intelligence,**32*(3), 478–500. 20075473 10.1109/TPAMI.2009.30

[CR15] Hessels, R. S., Hooge, I. T. C., & Kemner, C. (2016). An in-depth look at saccadic search in infancy. *Journal of Vision,**16*(8), 10. 27299770 10.1167/16.8.10

[CR16] Hessels, R. S., Niehorster, D. C., Kemner, C., & Hooge, I. T. C. (2017). Noise-robust fixation detection in eye movement data: Identification by two-means clustering (I2MC). *Behavior Research Methods,**49*(5), 1802–1823. 27800582 10.3758/s13428-016-0822-1 PMC5628191

[CR17] Hessels, R. S., Niehorster, D. C., Nyström, M., Andersson, R., & Hooge, I. T. C. (2018). Is the eye-movement field confused about fixations and saccades? a survey among 124 researchers. *Royal Society Open Science,**5*(8), 180502. 30225041 10.1098/rsos.180502 PMC6124022

[CR18] Hooge, I. T. C., Niehorster, D. C., Nyström, M., Andersson, R., & Hessels, R. S. (2018). Is human classification by experienced untrained observers a gold standard in fixation detection? *Behavior Research Methods,**50*(5), 1864–1881. 29052166 10.3758/s13428-017-0955-x PMC7875941

[CR19] Hooge, I. T. C., Niehorster, D. C., Nyström, M., Andersson, R., & Hessels, R. S. (2022). Fixation classification: How to merge and select fixation candidates. *Behavior Research Methods,**54*(6), 2765–2776. 35023066 10.3758/s13428-021-01723-1 PMC9729319

[CR20] Hoppe, S., & Bulling, A. (2016). End-to-End Eye Movement Detection Using Convolutional Neural Networks. arXiv:1609.02452.

[CR21] Huang, Z., Zhang, T., Heng, W., Shi, B., & Zhou, S. (2020) RIFE: Real-Time intermediate flow estimation for video frame interpolation. Nov. 2020.

[CR22] Kasneci, E., Kasneci, G., Kübler, T. C., & Rosenstiel, W. (2015). Online Recognition of Fixations, Saccades, and Smooth Pursuits for Automated Analysis of Traffic Hazard Perception. In P. Koprinkova-Hristova, V. Mladenov, & N. K. Kasabov (Eds.), *Artificial Neural Networks, Springer Series in Bio-/Neuroinformatics* (pp. 411–434). Cham. Springer International Publishing.

[CR23] Kassner, M., Patera, W., & Bulling, A. (2014). Pupil: An open source platform for pervasive eye tracking and mobile gaze-based interaction. In: Proceedings of the 2014 ACM international joint conference on pervasive and ubiquitous computing: Adjunct publication, UbiComp ’14 Adjunct, pages 1151–1160, New York, USA, Sept. 2014. Association for Computing Machinery.

[CR24] Kemner, C., van Ewijk, L., van Engeland, H., & Hooge, I. (2008). Brief report: Eye movements during visual search tasks indicate enhanced stimulus discriminability in subjects with PDD. *Journal of Autism and Developmental Disorders,**38*(3), 553–557. 17610058 10.1007/s10803-007-0406-0 PMC2254472

[CR25] Kinsman, T., Evans, K., Sweeney, G., Keane, T., & Pelz, J. (2012). Ego-motion compensation improves fixation detection in wearable eye tracking. In: Proceedings of the symposium on eye tracking research and applications, ETRA ’12, pages 221–224, New York, USA. Association for Computing Machinery.

[CR26] Klein, C. (2019). Eye Movement Research - An Introduction to its Scientific Foundations and Applications.

[CR27] Komogortsev, O. V., Gobert, D. V., Jayarathna, S., Koh, D. H., & Gowda, S. M. (2010). Standardization of automated analyses of oculomotor fixation and saccadic behaviors. *IEEE Transactions on Biomedical Engineering,**57*(11), 2635–2645. 10.1109/TBME.2010.2057429 20667803

[CR28] Komogortsev, O. V., & Karpov, A. (2012). Automated classification and scoring of smooth pursuit eye movements in the presence of fixations and saccades. *Behavior Research,**45*(1), 203–215. 10.3758/s13428-012-0234-9 22806708

[CR29] Komogortsev, O. V., & Khan, J. I. (2009). Eye movement prediction by oculomotor plant Kalman filter with brainstem control. *Journal of Control Theory and Applications,**7*(1), 14–22.

[CR30] Kothari, R., Yang, Z., Kanan, C., Bailey, R., Pelz, J. B., & Diaz, G. J. (2020). Gaze-in-wild: A dataset for studying eye and head coordination in everyday activities. *Scientific Reports,**10*(1), 2539. 32054884 10.1038/s41598-020-59251-5 PMC7018838

[CR31] Larsson, L., Nyström, M., Andersson, R., & Stridh, M. (2015). Detection of fixations and smooth pursuit movements in high-speed eye-tracking data. *Biomedical Signal Processing and Control,**18*, 145–152.

[CR32] Martinez-Conde, S., Macknik, S. L., & Hubel, D. H. (2004). The role of fixational eye movements in visual perception. *Nature Reviews Neuroscience,**5*(3), 229–240. 14976522 10.1038/nrn1348

[CR33] Nyström, M., & Holmqvist, K. (2010). An adaptive algorithm for fixation, saccade, and glissade detection in eyetracking data. *Behavior Research Methods,**42*(1), 188–204. 20160299 10.3758/BRM.42.1.188

[CR34] Olsen, A. (2012). The Tobii I-VT fixation filter. *Tobii Technology,**21*, 4–19.

[CR35] Otero-Millan, J., Troncoso, X. G., Macknik, S. L., Serrano-Pedraza, I., & Martinez-Conde, S. (2008). Saccades and microsaccades during visual fixation, exploration, and search: Foundations for a common saccadic generator. *Journal of vision,**8*(14), 21. 10.1167/8.14.21 19146322

[CR36] Patla, A. E., & Vickers, J. N. (1997). Where and when do we look as we approach and step over an obstacle in the travel path? *Neuroreport,**8*(17), 3661–3665. 9427347 10.1097/00001756-199712010-00002

[CR37] Pérez-Edgar, K., MacNeill, L. A., & Fu, X. (2020). Navigating through the experienced environment: Insights from mobile eye tracking. *Current Directions in Psychological Science,**29*(3), 286–292. 33642706 10.1177/0963721420915880 PMC7909451

[CR38] Rai, Y., Gutiérrez, J., & Le Callet, P. (2017). A dataset of head and eye movements for 360 degree images. In: Proceedings of the 8th ACM on multimedia systems conference, MMSys’17, pages 205–210, New York, USA, June 2017. Association for Computing Machinery.

[CR39] Rayner, K. (1978). Eye movements in reading and information processing. Psychological Bulletin, 85(3):618–660. 353867

[CR40] Rayner, K. (1978). Eye movements in reading and information processing. *Psychological Bulletin,**85*(3), 618–660. 353867

[CR41] Rayner, K. (1998). Eye movements in reading and information processing: 20 years of research. *Psychological Bulletin,**124*(3), 372–422. 9849112 10.1037/0033-2909.124.3.372

[CR42] Salvucci, D. D. & Goldberg, J. H. (2000) Identifying fixations and saccades in eye-tracking protocols. In: Proceedings of the 2000 symposium on eye tracking research & applications, ETRA ’00, pages 71–78, New York, USA. Association for Computing Machinery.

[CR43] Santini, T., Fuhl, W., Kübler, T., & Kasneci, E. (2016). Bayesian identification of fixations, saccades, and smooth pursuits. In: Proceedings of the ninth biennial ACM symposium on eye tracking research & applications, ETRA ’16, pages 163–170, New York, USA, Mar. 2016. Association for Computing Machinery.

[CR44] Schütz, A. C., Braun, D. I., & Gegenfurtner, K. R. (2011). Eye movements and perception: A selective review. *Journal of Vision,**11*(5), 9. 21917784 10.1167/11.5.9

[CR45] Sparks, D. L. (2002). The brainstem control of saccadic eye movements. *Nature Reviews Neuroscience,**3*(12), 952–964. 12461552 10.1038/nrn986

[CR46] Startsev, M., Agtzidis, I., & Dorr, M. (2019). 1D CNN with BLSTM for automated classification of fixations, saccades, and smooth pursuits. *Behavior Research Methods,**51*(2), 556–572. 30411227 10.3758/s13428-018-1144-2

[CR47] Startsev, M., Agtzidis, I., & Dorr, M. (2019). 1D CNN with BLSTM for automated classification of fixations, saccades, and smooth pursuits. *Behavior Research Methods,**51*(2), 556–572. 10.3758/s13428-018-1144-2 30411227

[CR48] Startsev, M., & Zemblys, R. (2022). Evaluating eye movement event detection: A review of the state of the art. *Behavior Research Methods,**55*, 1653–1714. 35715615 10.3758/s13428-021-01763-7

[CR49] Steil, J., Huang, M. X., & Bulling, A. (2018). Fixation detection for head-mounted eye tracking based on visual similarity of gaze targets. In: Proceedings of the 2018 ACM symposium on eye tracking research & applications, number Article 23 in ETRA ’18, pages 1–9, New York, USA, June 2018. Association for Computing Machinery.

[CR50] Tonsen, M., Baumann, C. K., Dierkes, K. (2020). A high-level description and performance evaluation of pupil invisible.

[CR51] van der Lans, R., Wedel, M., & Pieters, R. (2011). Defining eye-fixation sequences across individuals and tasks: The Binocular-Individual Threshold (BIT) algorithm. *Behavior Research Methods,**43*(1), 239–257. 21287116 10.3758/s13428-010-0031-2 PMC3048294

[CR52] Veneri, G., Piu, P., Rosini, F., Federighi, P., Federico, A., & Rufa, A. (2011). Automatic eye fixations identification based on analysis of variance and covariance. *Pattern Recognition Letters,**32*(13), 1588–1593.

[CR53] Wan, Q., Kaszowska, A., Panetta, K., Taylor, H.A., & Agaian, S. (2019). A comprehensive head-mounted eye tracking review: Software solutions, applications, and challenges. *Electronic Imaging, 2019*(3):654–1–654–9.

[CR54] Zagoruyko, S., & Komodakis N. (2015). Learning to Compare Image Patches via Convolutional Neural Networks. pages 4353–4361.

[CR55] Zemblys, R. (2017). Eye-movement event detection meets machine learning. Biomedical Engineering, *20*(1).

[CR56] Zemblys, R., Niehorster, D. C., & Holmqvist, K. (2019). Gazenet: End-to-end eye-movement event detection with deep neural networks. *Behavior Research Methods,**51*(2), 840–864. 30334148 10.3758/s13428-018-1133-5

[CR57] Zemblys, R., Niehorster, D. C., Komogortsev, O., & Holmqvist, K. (2018). Using machine learning to detect events in eye-tracking data. *Behavior Research Methods,**50*(1), 160–181. 28233250 10.3758/s13428-017-0860-3

