# Supplementary material for: Strategies for enhancing automatic fixation detection in head-mounted eye tracking
Source: Behav Res Methods. 2024 Apr 9;56(6):6276–98. doi: 10.3758/s13428-024-02360-0 (PMC11541274; doi:10.3758/s13428-024-02360-0)
Supplement: Supplementary file 4 — (pdf 807 KB) [file 13428_2024_2360_MOESM4_ESM.pdf]

I-XT	grid values	algorithm variants
v_{thr}	300 – 1500 px/s; step: 100 px/s	all velocity-based (V)
d_{thr}	10 – 40 px; step: 2.5 px	all dispersion-based (D)
a_{thr}	0 – 2 °; step: 0.25 °	+F
t_{thr}	0 – 120 ms; step: 10 ms	+F
d_{min}	0 – 120 ms; step: 10 ms	+F
gain (velocity)	0 – 1.5; step: 0.1	I-VAT+F^{rel}
gain (dispersion)	0 – 0.05; step: 0.05	I-DAT+F^{rel}
window size	55 – 405 ms; step: 50 ms	I-(V/D)AT+F^{rel}
REMoDNaV		
noise_factor	0 – 10; step: 1	all
velthresh_startvelocity	[100 °/s, 300 °/s]	all
min_fixation_duration	0 – 120 ms; step: 20 ms	all
max_initial_saccade_freq	2 – 10 Hz; step: 4 Hz	all
saccade_context_window	[1 s, 2 s]	all
savgol_length	[19 ms, 39 ms, 49 ms, 59 ms]	all
median_filter_length	[50 ms, 100 ms]	all
Steil et al.		
similarity threshold	1.1 – 1.4; step: 0.025, and [0.7, 0.9]	both
crop size	50 px – 300 px; step: 50 px	Steil et al.+F
a_{thr}	0 – 2 °; step: 0.25 °	Steil et al.+F
t_{thr}	0 – 120 ms; step: 10 ms	Steil et al.+F
d_{min}	0 – 120 ms; step: 10 ms	Steil et al.+F

Supplementary Table 1

Gridscan parameters

Gridscan parameters for all algorithms and variants tested. Some parameters are valid only for specific algorithm variants, as indicated by the last column. Velocity thresholds were implemented in units of px/ s, which can be converted to °/ s using the field of view of the eye tracker.

.

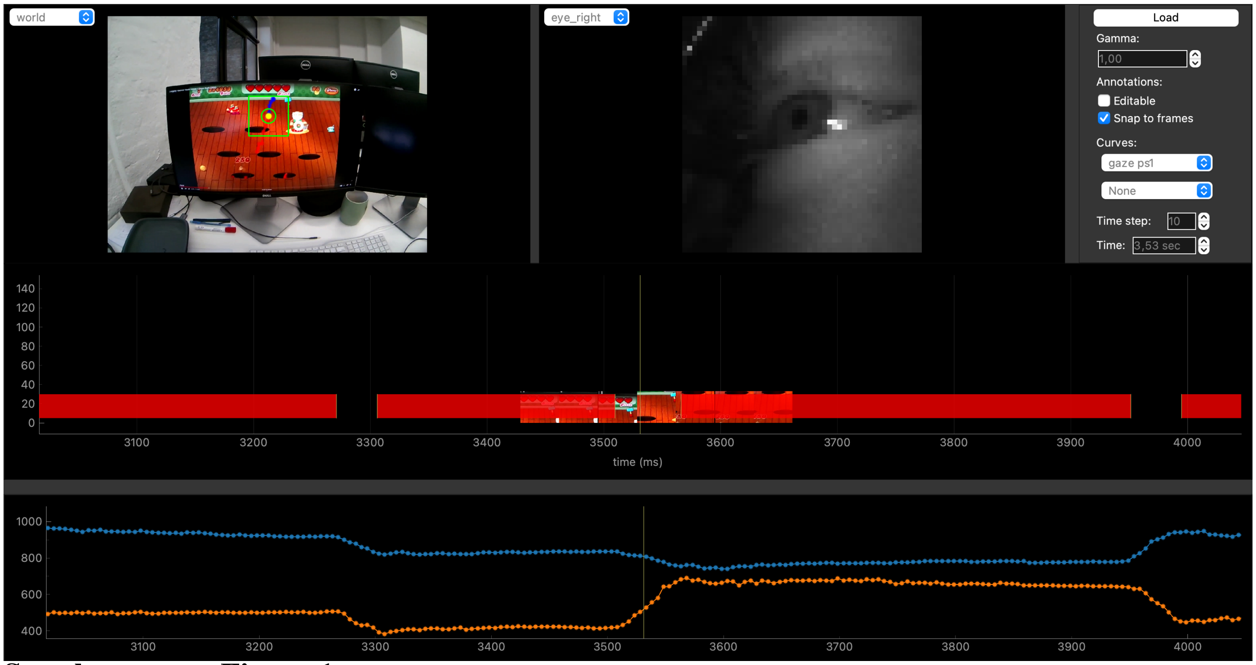

Supplementary Figure 1

Screenshot of annotation software

Screenshot of the custom software used to annotate ground-truth fixations. The software was designed so that the annotator had a view of the scene camera with the current gaze point and a trace of past and future gaze points superimposed, a view of one of the eye camera images, and gaze predictions all in the same window. Navigation forward and backward in time was implemented via key strokes or dragging with the mouse in the central widget, where annotated fixations were visualized as red bars.
